# Movement maintains forebrain neurogenesis via peripheral neural feedback in larval zebrafish

**DOI:** 10.7554/eLife.31045

**Published:** 2018-03-12

**Authors:** Zachary Jonas Hall, Vincent Tropepe

**Affiliations:** 1 Department of Cell & Systems BiologyUniversity of TorontoTorontoCanada; Max Planck Institute for Heart and Lung ResearchGermany

**Keywords:** neurogenesis, brain, zebrafish, motor experience, dorsal root ganglia, neural stem cell, Zebrafish

## Abstract

The postembryonic brain exhibits experience-dependent development, in which sensory experience guides normal brain growth. This neuroplasticity is thought to occur primarily through structural and functional changes in pre-existing neurons. Whether neurogenesis also mediates the effects of experience on brain growth is unclear. Here, we characterized the importance of motor experience on postembryonic neurogenesis in larval zebrafish. We found that movement maintains an expanded pool of forebrain neural precursors by promoting progenitor self-renewal over the production of neurons. Physical cues associated with swimming (bodily movement) increase neurogenesis and these cues appear to be conveyed by dorsal root ganglia (DRG) in the zebrafish body: DRG-deficient larvae exhibit attenuated neurogenic responses to movement and targeted photoactivation of DRG in immobilized larvae expands the pallial pool of proliferative cells. Our results demonstrate the importance of movement in neurogenic brain growth and reveal a fundamental sensorimotor association that may couple early motor and brain development.

## eLife digest

Sensory experiences early in life help the brain to grow and develop. For example, raising animals in complete darkness stops the visual areas of their brain from forming properly. Previous studies have shown that sensory input helps to strengthen the connections between already existing brain cells, but it is unclear if it affects the actual creation of new brain cells.

Conditions that reduce the mobility of young children, such as muscular disease, are often accompanied by learning difficulties. This suggests that physical movement may be important for healthy brain development. Scientists have previously found a link between exercise and an increased production of new brain cells in adults. However, such a link has not been established earlier in life, when the brain is developing the most.

To address this, Hall and Tropepe studied how movement affects the brain development in zebrafish larvae, at an age when many of their brain cells are created. Restraining the larvae decreased their physical movement, while making them swim against a current increased their movement. Hall et al. looked at how this affected the larvae’s number of so called progenitor cells – the cells from which brain cells are created. When the larvae moved less, the number of progenitor cells decreased. But when they moved more frequently, the amount of progenitor cells increased.

The experiments also showed that some sensory cells, which detect sensations associated with movement of the body during swimming, are linked to brain cell production. Blocking the development of these sensory cells prevented the rise in progenitor cells seen with increased movement in the larvae. However, activating these sensory cells in immobilised larvae increased the number of progenitor cells, even though the larvae could not move. These findings suggest that measures to increase physical movement in young children could be used to help the brain develop normally.

## Introduction

During postembryonic development, the brain begins processing sensory information from the environment for the first time and continues to grow, exhibiting elevated levels of neuroplasticity compared to later stages of life. The combination of these factors makes postembryonic brain development highly susceptible to sensory experience (Knudsen, 2004). This susceptibility to experience is evident in the ‘critical’ and ‘sensitive’ periods early in life, in which sensory experiences drive permanent or near permanent changes in brain structure and function, respectively (Knudsen, 2004). Historically, neuroplastic changes associated with early sensory experience were thought to be restricted to structural and functional changes in pre-existing neurons, such as visual experience-dependent synaptic remodeling in thalamocortical projections associated with the development of ocular dominance (Coleman et al., 2010). However, neurogenesis persists postembryonically throughout the brain, either ceasing or curtailing in adolescence or adulthood (Lindsey and Tropepe, 2006). Some neuronal populations appear to be uniquely generated during postembryonic development (Wei et al., 2011), and this process may be regulated by sensory experience (He et al., 2014). Thus, neurogenesis may also mediate the effects of early experience on brain development.

Outside of postembryonic development, one of the best-characterized models of experience-dependent regulation of neurogenesis is the increase in cell proliferation in the subgranular zone (SGZ) of the dentate gyrus in the adult mammalian hippocampus (HP) following periods of aerobic running exercise (van Praag et al., 1999a). Since its initial discovery, studies have gone on to link exercise-induced neurogenesis to improvements in cognition, such as spatial learning (van Praag et al., 1999b) and cognitive flexibility (Anacker and Hen, 2017). Studies have also incorporated exercise as a therapeutic intervention to combat neuropsychiatric disorders associated with impaired neurogenesis (Vakhrusheva et al., 2016; Kandola et al., 2016). However, whether physical activity affects forebrain neurogenesis during postembryonic development, when animals first gain control of their movements while exhibiting elevated levels of neurogenesis throughout the brain compared to adulthood, remains unexplored. Furthermore, such a relationship between movement and forebrain growth early in development may help explain the positive correlation between physical activity and cognitive function reported in human children (Tomporowski et al., 2008; Best, 2010).

Here, we sought to investigate the relationship between movement and neurogenesis in larval zebrafish during a developmental period in which they first begin to exhibit voluntary movements (Buss and Drapeau, 2001), have brains and peripheral nervous systems sufficiently developed to process sensory input, and continue to exhibit elevated rates of neurogenesis in many brain divisions compared to adulthood (Lindsey and Tropepe, 2006; Feliciano et al., 2015). In addition to the well-documented advantages of larval zebrafish as models for genetic and pharmacological tractability, here we also use them for our ability to control the sensory experiences of larvae, enabling the isolation of different sensory cues associated with movement to identify the nature of sensory feedback driving neurogenic change. We first developed paradigms to both reduce and increase swimming behaviour in larvae and sample for changes in neurogenesis in the forebrain. We then sought to isolate the different sensory cues associated with movement and identify which cue drives a neurogenic response in the forebrain. Finally, we tested whether dorsal root ganglia (DRG), sensory neurons that convey mechanical sensations from the zebrafish trunk, are required to mediate movement-dependent neurogenesis. We did this by both pharmacologically generating larvae deficient in DRG along the trunk and by stimulating the DRG via photoactivation of ankyrin-containing transient receptor potential channels (TRPA1b) in completely immobilized larvae. Altogether, we present a novel and robust relationship between movement and postembryonic forebrain neurogenesis, demonstrating that neural feedback associated with physical movement may provide a simple mechanism through which motor and brain development become coupled early in life.

## Results

All statistical tests are preceded by a superscript numeral that can be used to reference that test in our calculations of statistical powers, summarized in Table 1.

**Table 1.** Statistical powers derived from posthoc power calculations for all major findings. All power analyses were performed using G*Power (Faul et al., 2007).

Statistical test	Power (1-β error probability)
1	0.999
2	1.000
3	1.000
4	0.983
5	0.159
6	0.053
7	0.939
8	0.840
9	0.999
10	0.076
11	0.110
12	0.956
13	0.886
14	0.062
15	0.998
16	0.814
17	0.060
18	0.101
19	0.164
20	0.918
21	0.992
22	0.383
23	0.683
24	0.226
25	0.702
26	0.212
27	0.600
28	0.052
29	0.910
30	0.420
31	0.075
32	0.050
33	1.000
34	0.836
35	0.603
36	1.000
37	1.000
38	1.000
39	0.585
40	0.575

### Movement restraint reduces swimming episodes without impairing swimming ability

**Figure 1.**
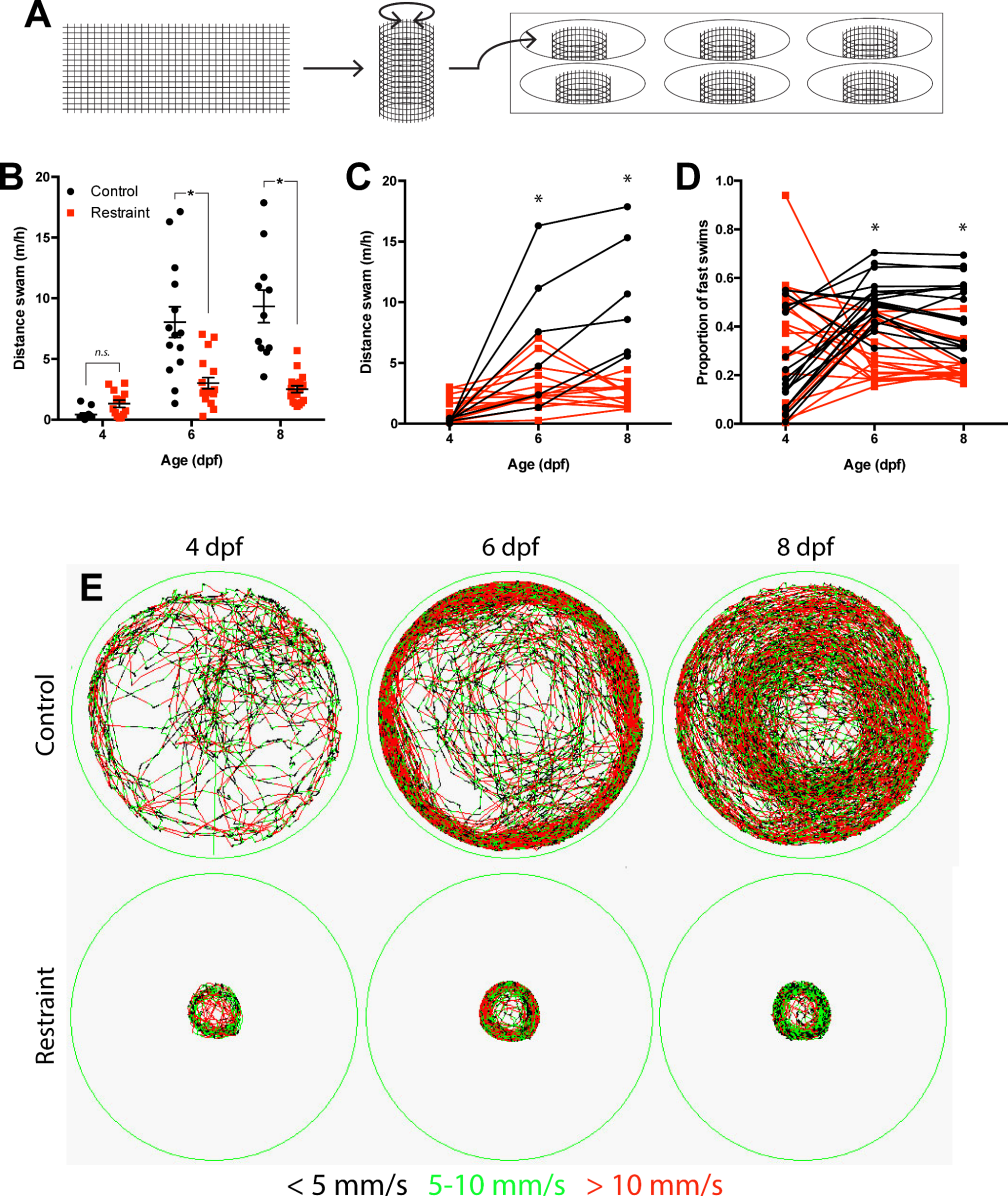
Movement restraint reduces swimming. (**A**) Apparatus used to restrain movement in zebrafish larvae by reducing the volume of water available to the larvae to swim using a mesh barrier. Movement restraint reduced swimming on 6 to 8 dpf both between groups (**B**; control: n_4,6,8 dpf_ = 12, 14, 11; restraint, n_4,6,8 dpf_ = 13, 18, 18; Data are represented as mean ± SEM) and within individual larvae (**C**; control n = 6; restraint n = 13). Movement restraint also reduced the proportion of fast swims (>10 mm/s) exhibited by larvae by 6–8 dpf (**D**; n = 18). (**E**) Representative traces from 1 hr time-bins for recorded larvae from 4 to 8 dpf. Colour coding represents movement speed. *p<0.05.

We first established a paradigm through which we could control the amount of swimming exhibited by zebrafish larvae noninvasively. We used movement restraint, in which larvae were confined to a smaller portion of 6-well plates by a mesh cylinder (Figure 1A) and tested if such restraint would reduce swimming from 3 to 9 days post fertilization (dpf). Movement restraint significantly reduced the hourly distance swam by 6 and 8 dpf in both non-repeated (Figure 1B; Treatment x Age Interaction: ^1^F_2,80_ = 14.08, p<0.01) and repeated (Figure 1C; Video 1; Treatment x Age Interaction: ^2^F_2,34_ = 14.16, p<0.01) experimental designs compared to unrestrained controls. Furthermore, movement restraint also prevented the increase in the proportion of fast swims (>10 mm/s) first exhibited by control larvae on 6 dpf (Figure 1D–E; Treatment x Age Interaction: ^3^F_2,68_ = 14.90, p<0.01).

**Figure 1—figure supplement 1.**
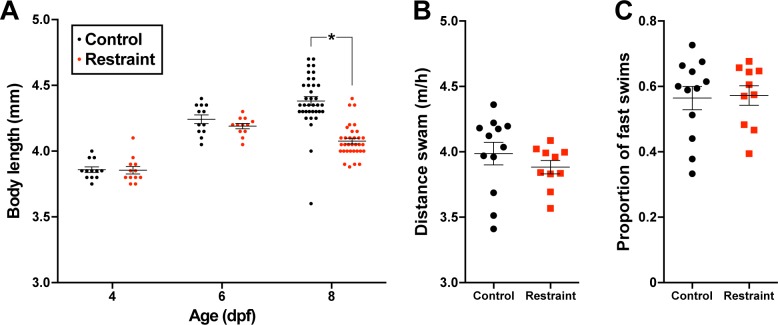
Body lengths of zebrafish larvae reared under physical restraint or control conditions on 3 dpf (n = 12), 6 dpf (control n = 12, restraint n = 12), and 9 dpf (control n = 36, restraint n = 33). Hourly distance swam (B; control n = 12, restraint n = 10) and proportion of fast swims (>10 mm/s, C; control n = 12, restraint n = 10) in unrestrained wells by larvae reared previously under restraint or control conditions. Data are represented as mean ± SEM.

**Video 1.** Video recorded using Zebralab software (Viewpoint) of 6 dpf zebrafish larvae swimming in restraint (above) or control (below) wells.

Because of the possibility that chronic movement restraint may impair larval development, we sampled body length of control and restrained larvae throughout the restraint period. We found that movement restraint did not affect larval body length by 6 dpf, but reduced body length by 9 dpf (Figure 1—figure supplement 1A; ^4^F_2,111_ = 13.10, p<0.01). To test if this reduction in body length affected motor ability, we repeated our movement restraint paradigm and, on 8 dpf, we moved restraint larvae into control wells to record unrestrained swimming behaviour. Prior movement restraint did not impair either hourly distance swam (Figure 1—figure supplement 1B; ^5^t_20_ = 0.98, p=0.34) or proportion of fast swims (Figure 1—figure supplement 1C, ^6^t_20_ = 0.16, p=0.87) in unrestrained conditions. Collectively, we found that our restraint paradigm reduced motor experience in larvae by 6 dpf without impairing swimming ability.

### Movement restraint promotes cell differentiation over self-renewal in forebrain neural precursor populations

**Figure 2.**
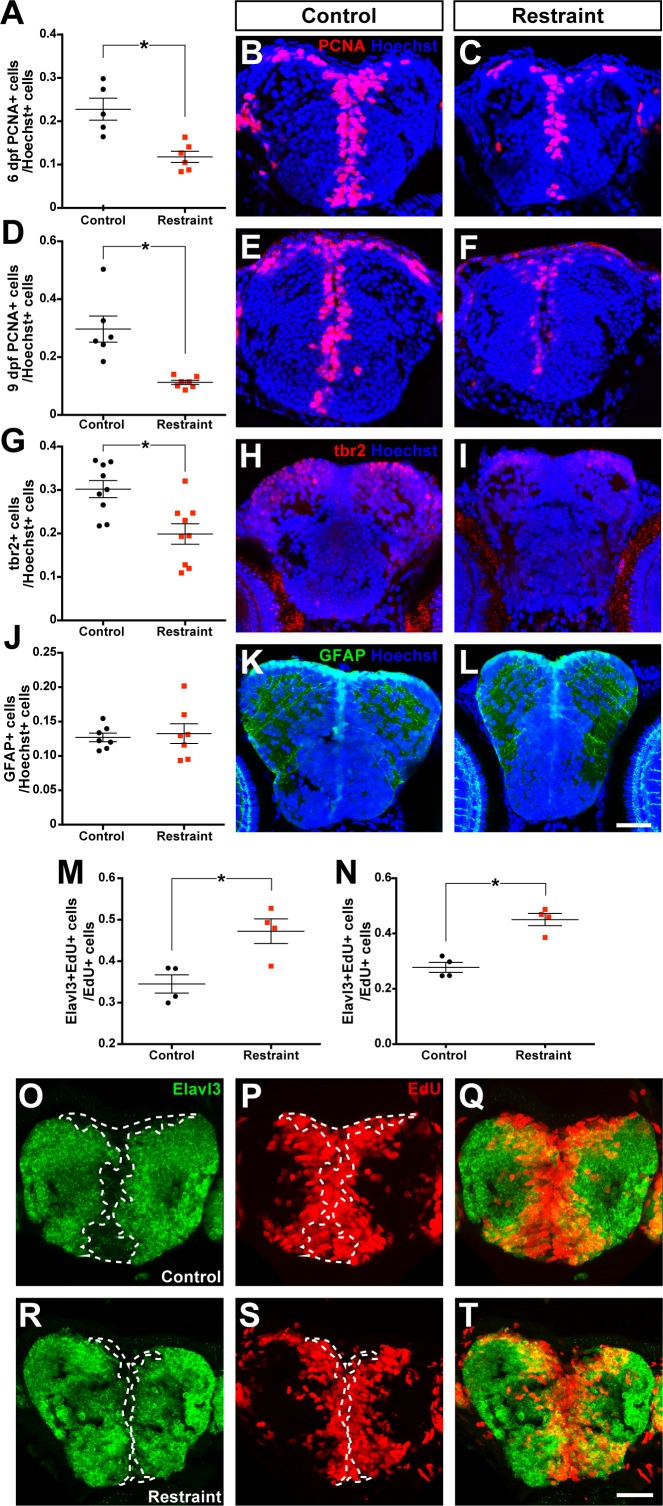
Movement restraint reduces cell proliferation in the larval forebrain. By 6 dpf, movement restraint reduces the proportion of PCNA+ cells in the forebrain (A-C; control n = 5, restraint n = 6). This reduction in PCNA +cells is maintained when movement restraint is continued until 9 dpf (D-F; control n = 6, restraint n = 7). Movement restraint until 9 dpf also reduces tbr2+ cells in the pallium (G-I; n = 9) without affecting the number of GFAP+ radial glia stem cells in the pallium (J-L; n = 7; scale bar for micrographs in B-L = 30 µm). Following a 24 hr pulse with EdU starting on 5 dpf, fewer EdU+ cells in the subpallium (**M**) and pallium (N; n = 4) co-label for the neuronal fate marker Elavl3 in controls (**O–Q**) compared to movement restrained larvae (R-T; scale bar = 20 µm). White dotted lines mark the boundaries of Elavl3+ expression to highlight the increased overlap between EdU+ cell cohorts and Elavl3+ in restrained larvae. *p<0.05. Data are represented as mean ± SEM.

To test for changes in neurogenesis in the restrained larval zebrafish brain, we sampled the proportion of PCNA+ cells in the pallium, subpallium, olfactory bulb, and optic tectum of 6 dpf larval zebrafish. Movement restraint significantly reduced the proportion of proliferative (PCNA+) cells in the forebrain of zebrafish by 6 dpf (Figure 2A–C; ^7^t_9_ = 4.07, p<0.01) sampled across consecutive coronal sections (Figure 2—figure supplement 1), without affecting forebrain size (Table 2). This difference was attributed to a reduction in the proportion of PCNA+ cells in both the subpallium (Figure 2—figure supplement 2A; ^8^t_5_ = 3.77, p=0.01) and pallium (Figure 2—figure supplement 2B; ^9^t_5_ = 7.36, p<0.01). Conversely, movement restraint did not affect the size (Table 2) or proportion of PCNA+ cells in the olfactory bulb (Figure 2—figure supplement 2C; ^10^t_9_ = 0.53, p=0.61) or optic tectum (Figure 2—figure supplement 2D; ^11^t_8_ = 0.87, p=0.41) by 6 dpf. Movement restraint reduced forebrain size by 9 dpf (Table 2); however, after correcting for forebrain size, chronic restraint still significantly reduced the proportion of PCNA+ cells in the forebrain by 9 dpf compared to controls (Figure 2D–F; ^12^U = 0, p<0.01). Movement restraint also reduced the proportion of tbr2+ cells, a protein marker of intermediate progenitors and newly generated neurons (Englund et al., 2005), in the pallium by 9 dpf (Figure 2G–I; ^13^t_16_ = 3.37, p<0.01) without affecting the proportion of pallial GFAP+ radial neural stem cells in *Tg(GFAP:gfp)* embryos (Figure 2J–L; ^14^t_12_ = 0.35, p=0.73). Thus, movement restraint reduced the size of the pool of proliferative cells, presumably neural progenitors, in the forebrain specifically, without affecting the size of the resident radial stem cell population.

**Figure 2—figure supplement 1.**
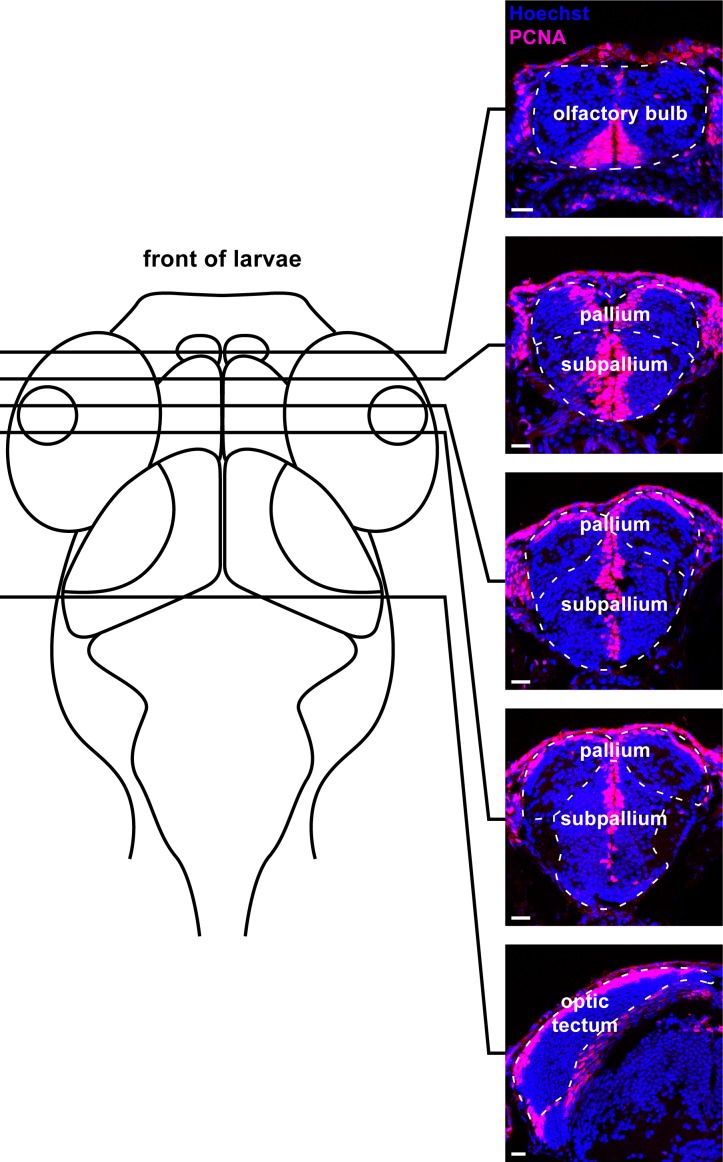
Example traces of brain regions sampled through coronal sections in the larval zebrafish brain. Micrographs (20 µm thickness) with example boundaries traced for the olfactory bulb, pallium, subpallium, and optic tectum (white dotted line) along with their approximate rostrocaudal position on a schematic of a dorsal view of the larval zebrafish head. Scale bars = 20 µm.

**Figure 2—figure supplement 2.**
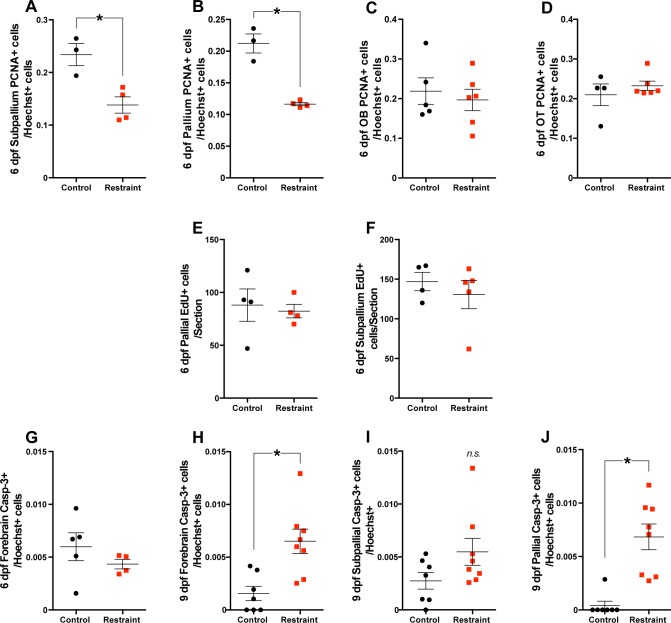
Movement restraint reduces the number of PCNA+ cells in the subpallium (**A**) and pallium (B; control n = 3; restraint n = 4) of 6 dpf zebrafish larvae compared to unrestrained controls. Movement restraint did not affect the number of PCNA +cells in the olfactory bulb (OB; C; control n = 5, restraint n = 6) or optic tectum (OT; D; control n = 4, restraint n = 6) on 6 dpf. Movement restraint did not affect the number of EdU+ cells produced in the pallium (E; n = 4) or subpallium (F; control n = 4, restraint n = 5) over 24 hr from 5 to 6 dpf. Movement restraint did not affect the number of activated caspase-3+ (Casp3) cells in the zebrafish forebrain on 6 dpf (G; control n = 5, restraint n = 4) and increased the number of Casp3+ cells in the forebrain by 9 dpf (H; control n = 7, restraint n = 8): this effect was not found in the subpallium (I; control n = 7, restraint n = 8) and was specific to the pallium (J; control n = 7, restraint n = 8). Data are represented as mean ± SEM.

**Table 2.** Changes in brain regions (sampled as Hoechst + cells/section following the procedures outlined underneath the ‘Cell Counting’ subheading in the Materials and methods) sampled across experiments. All power analyses were performed using G*Power (Peirce, 2008).

Experiment	Region sampled (Hoechst + cells/Section)	Significantly different?	Test statistic	P value	Power (1-β error probability)
Restraint (3–6 dpf)	Forebrain	No	t_9_ = 1.018	0.3351	0.104
	Pallium	No	t_5_ = 0.4206	0.6915	0.054
	Subpallium	No	t_5_ = 1.685	0.1528	0.280
	Olfactory Bulb	No	t_7_ = 0.3330	0.7489	0.060
	Optic tectum	No	t_8_ = 1.664	0.1347	0.300
Restraint (3–9 dpf	Forebrain	**Yes (Control > Restraint)**	t_11_ = 3.890	0.0025	0.938
	Pallium	**Yes (Control > Restraint)**	t_13_ = 2.657	0.0198	0.704
	Subpallium	**Yes (Control > Restraint)**	t_23_ = 4.725	<0.0001	0.995
Exercise (3–6 dpf)	Pallium	No	t_8_ = 1.260	0.2430	0.199
	Subpallium	No	t_7_ = 1.435	0.1943	0.240
Exercise (3–9 dpf)	Pallium	No	t_15_ = 1.559	0.1397	0.310
	Subpallium	No	t_19_ = 0.2109	0.8352	0.055
Physical vs. Visual stimulation (3–6 dpf)	Pallium	No	F_2,14_ = 0.5679	0.5792	0.122
	Subpallium	No	F_2,14_ = 0.2594	0.7751	0.082
AG1478 vs. DMSO (3 dpf)	Pallium	No	t_7_ = 1.184	0.2751	0.170
AG1478 vs. DMSO (6 dpf)	Pallium	No	F3,79 = 1.852	0.1445	0.484

We then asked how movement restraint results in a reduced forebrain proliferative cell population. We reasoned that a reduction in this cell population might occur when either proliferative cells generate more differentiated cells at the expense of self-renewal, reducing the size of the proliferative population over successive divisions, or by apoptosis in the proliferative population. To sample cell differentiation in these forebrain populations, we exposed larvae to 5 mM 5-Ethynyl-2'-deoxyuridine (EdU), a synthetic thymidine analog that is incorporated in dividing cells, for 24 hr starting on 5 dpf, then sampled the proportion of EdU+ cells that also express Elavl3 protein, a marker of cells with a differentiated neuronal fate (Lindsey et al., 2012). If movement restraint biased the production of differentiating cells over progenitor self-renewal, we would predict that restrained larvae would exhibit more EdU+ cells that co-expressed Elavl3. Movement restraint significantly increased the proportion of newly generated cells that co-label with Elavl3 in both the pallium (Figure 2M; ^15^t_6_ = 6.02, p<0.01) and subpallium (Figure 2N–T; ^16^t_6_ = 3.43, p=0.01) without affecting the absolute number of EdU+ cells produced in the pallium (Figure 2—figure supplement 2E; ^17^t_6_ = 0.35, p=0.74) or subpallium (Figure 2—figure supplement 2F; ^18^t_7_ = 0.73, p=0.49). Conversely, movement restraint did not affect the number of cells expressing the apoptotic marker activated caspase-3 (Casp3) in the forebrain (Figure 2—figure supplement 2G; ^19^t_7_ = 1.06, p=0.32) by 6 dpf. By 9 dpf, however, movement restraint significantly increased the number of activated Casp3+ cells in the forebrain (Figure 2—figure supplement 2H; ^20^t_13_ = 3.56, p<0.01). This increase in apoptosis at 9 dpf was specific to the pallium (Figure 2—figure supplement 2I; ^21^U = 1, p<0.01) and not found in the subpallium (Figure 2—figure supplement 2J; ^22^t_13_ = 1.76, p=0.10). Despite this increase in pallial apoptosis, cell death rates remained low by 9 dpf and Casp3+ cells were not observed along the midline or dorsal surface of the brain, where the neurogenic niche lies. Together, these findings suggest that movement restraint biased newly generated cells to differentiate into neurons, ultimately at the expense of self-renewal.

### Rearing larvae against a strong current increases the size of the pallial proliferative cell population

**Figure 3.**
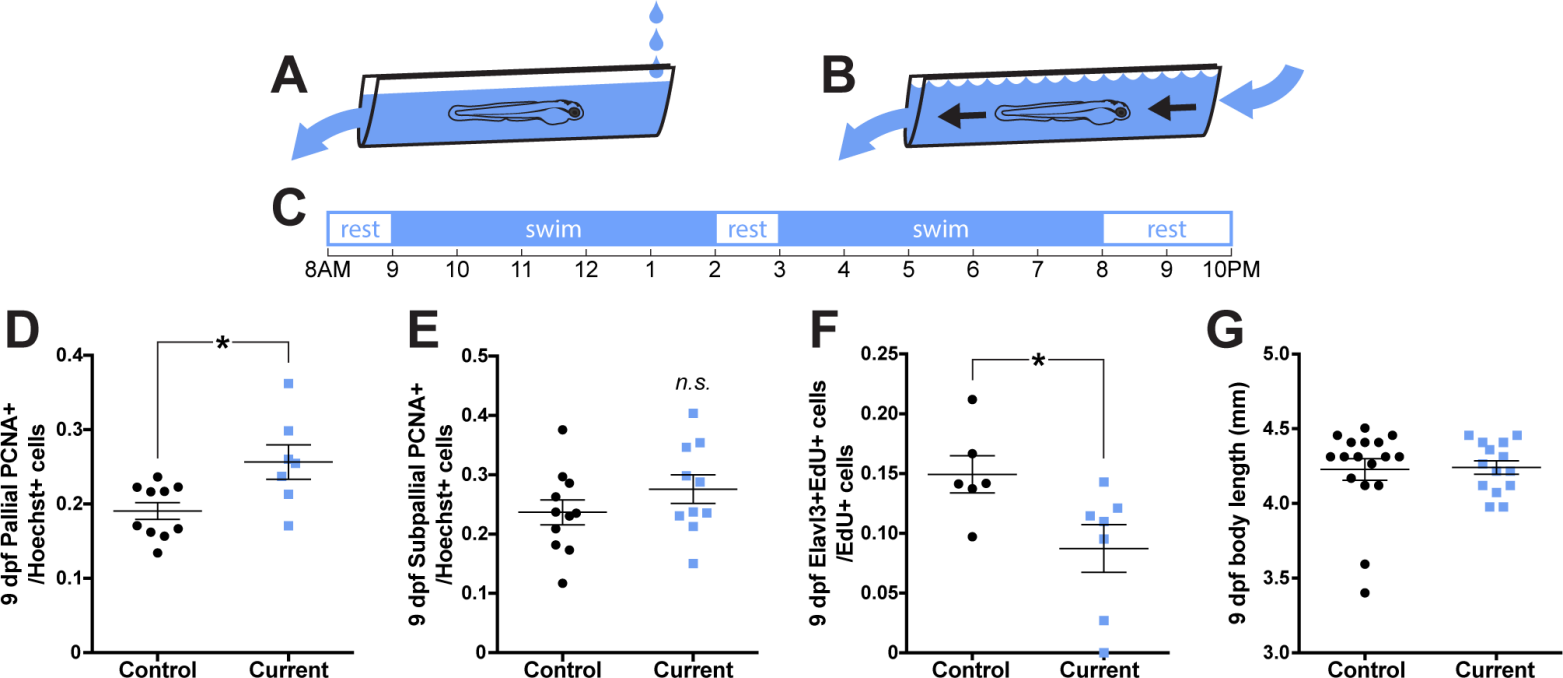
Rearing larvae against a displacing current increases pallial cell proliferation. From 3 dpf, larvae were reared in groups in a plastic canal with water flow producing (**A**) a weak current that did not displace larvae or (**B**) a strong current that would displace larvae on a daily schedule (**C**). By 9 dpf, larvae reared against a strong current exhibited significantly more PCNA +cells in the pallium (D; control n = 10, current n = 7), but not subpallium (E; control n = 11, current n = 10) compared to controls. Larvae reared against a strong current exhibited significantly less Elavl3/EdU co-labeling compared to controls (F; n = 6) and did not differ in body length from controls (G; control n = 17, current n = 14). *p<0.05. n.s. = not significant. Data are represented as mean ± SEM.

Because physical restraint may restrict more than just movement (i.e., reducing sensory input in a smaller space), we tested whether increasing movement could also impact forebrain cell proliferation. We raised larvae in groups (n = 15–20) housed in transparent plastic canals against different strengths of water current. Control larvae experienced no displacing current (Figure 3A; water dripping in and out, current did not displace larvae) and ‘exercised’ larvae experienced a strong current (Figure 3B; water flow strong enough to displace larvae) from 3 to 9 dpf following a daily schedule (Figure 3C). In the current condition, larvae would have to swim to counteract the flow of water and maintain their position in the canal, akin to forced exercise paradigms in rodents (Leasure and Jones, 2008). On 9 dpf, larvae reared against a strong current exhibited a greater proportion of PCNA+ cells in the pallium (Figure 3D; ^25^t_15_ = 2.80, p=0.01), but not the subpallium (Figure 3E; ^26^t_19_ = 1.22, p=0.24). On 9 dpf, the size of both brain regions was not affected by rearing treatment (Table 2). When we sampled pallial proliferation in larvae earlier, at 6 dpf, we again found an increase in the proportion of PCNA+ cells in the pallium in larvae reared against a strong current (Figure 3—figure supplement 1A; ^23^t_7_ = 2.76, p=0.03), while the subpallium was unaffected (Figure 3—figure supplement 1B; ^24^t_6_ = 1.27, p=0.25). Again, the size of both brain regions was not affected by rearing in a strong current on 6 dpf (Table 2). To test if increased movement affected cell differentiation as in our restraint paradigm, we exposed larvae to EdU in a petri dish overnight for 13 hr from 8 to 9 dpf prior to being returned to their swimming canals for a final 5 hr of current-rearing. Rearing larvae against a current reduced the number of newly generated (EdU+) cells that also expressed Elavl3 compared to controls (Figure 3F; ^27^t_11_ = 2.39, p=0.04), consistent with increased movement maintaining an expanded proliferative cell population over the generation of differentiated neurons. Rearing larvae against a current from 3 to 9 dpf did not affect body length (Figure 3G, ^28^U = 102, p=0.5081), suggesting these effects on pallial neurogenesis are not a product of overall growth. Together with our movement restraint data, the increase in pallial cell proliferation following exercise suggests that motor experience regulates forebrain neurogenesis specifically in the pallium, similar to the neurogenic effect of exercise in the mammalian SGZ.

**Figure 3—figure supplement 1.**
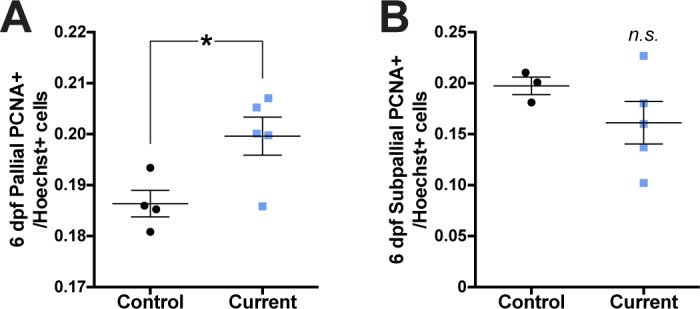
Rearing larvae against a strong current increased the proportion of PCNA+ cells in the pallium. By 6 dpf, larvae reared against a strong current exhibited significantly more PCNA+ cells in the pallium (A; control n = 4, current n = 5), but not subpallium (B; control n = 3, current n = 4) compared to controls. *p<0.05. n.s. = not significant. Data are represented as mean ± SEM.

### Movement-dependent maintenance of pallial cell proliferation requires physical, not visual, input associated with movement

**Figure 4.**
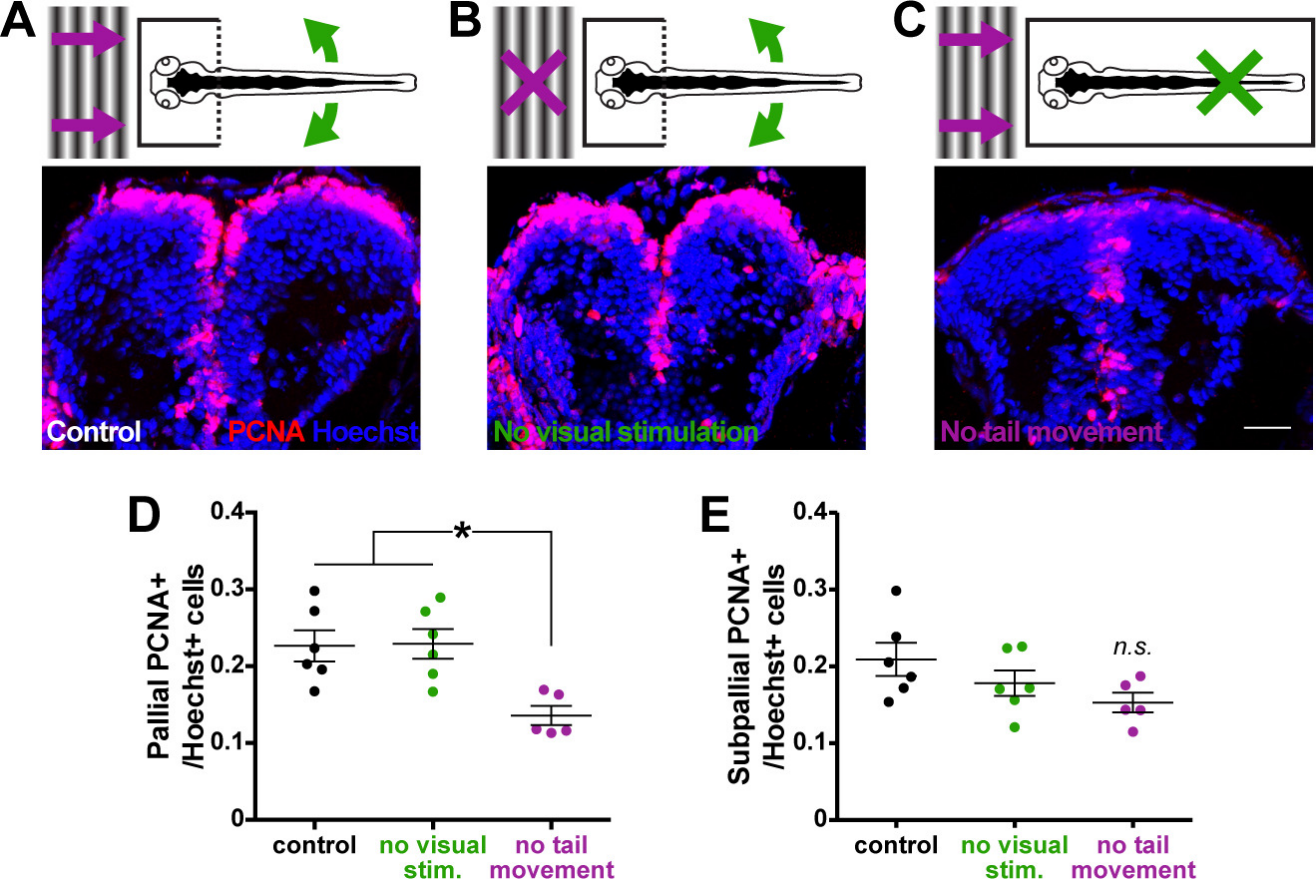
Tail movement, not visual stimulation, associated with locomotion maintains pallial cell proliferation. Larvae were fully embedded in agarose at 3 dpf and reared able to perceive either (**A**) visual stimulation associated with movement (a moving gradient; purple arrows) and tail movement (tail cut free from agarose; green arrows), (**B**) tail movement only, or (C; scale bar = 20 µm) visual stimulation only. By 6 dpf, larvae capable of tail movement exhibited more PCNA+ cells in the pallium (**D**) compared to larvae perceiving only visual stimulation associated with movement. Isolating visual or physical cues of movement had no significant affect on PCNA+ cell counts in the subpallium (E; control n = 6, physical only n = 6, visual only n = 5) by 6 dpf. *p<0.05. n.s. = not significant. Data are represented as mean ± SEM.

Upon establishing a link between motor experience and pallial neurogenesis, we asked if we could identify the modality of sensory feedback associated with movement driving cell proliferation. To isolate visual and physical components of movement, we restrained larvae entirely in agarose from 3 to 6 dpf, preventing locomotion. We then re-introduced visual stimulation associated with movement (optic flow) by exposing immobilized larvae to computer-generated visual gratings to simulate visual motion. Physical input associated with movement was re-introduced to immobilized larvae by cutting the tail of larvae free from agarose embedding, enabling swimming tail movement without bodily displacement in the environment. Control larvae were provided both visual stimulation (gratings) and tail movement (Figure 4A), whereas treatment groups experienced only either tail movement (Figure 4B) or visual stimulation (Figure 4C). Blocking tail movement (complete immobilization) significantly reduced the proportion of PCNA+ cells in the pallium by 6 dpf (Figure 4D; ^29^F_2,14_ = 7.89, p<0.01). Conversely, removing just visual stimulation had no impact on the number of PCNA+ cells in the pallium. Neither removing visual stimulation nor tail movement affected the proportion of PCNA+ cells in the subpallium (Figure 4E; ^30^F_2,14_ = 2.42, p=0.13).

Because immobilization could impair brain growth globally, we also sampled the number of Hoechst+ cells per section as a proxy for absolute forebrain size. Immobilization did not reduce the total number of cells in the pallium or subpallium (Table 2), instead affecting the PCNA+ cell population specifically. These results suggest that physical input associated with locomotion, specifically tail movement during swimming, drives changes in pallial neuroproliferation.

### Ablation of the lateral line does not impact pallial neuroproliferation

**Figure 5.**
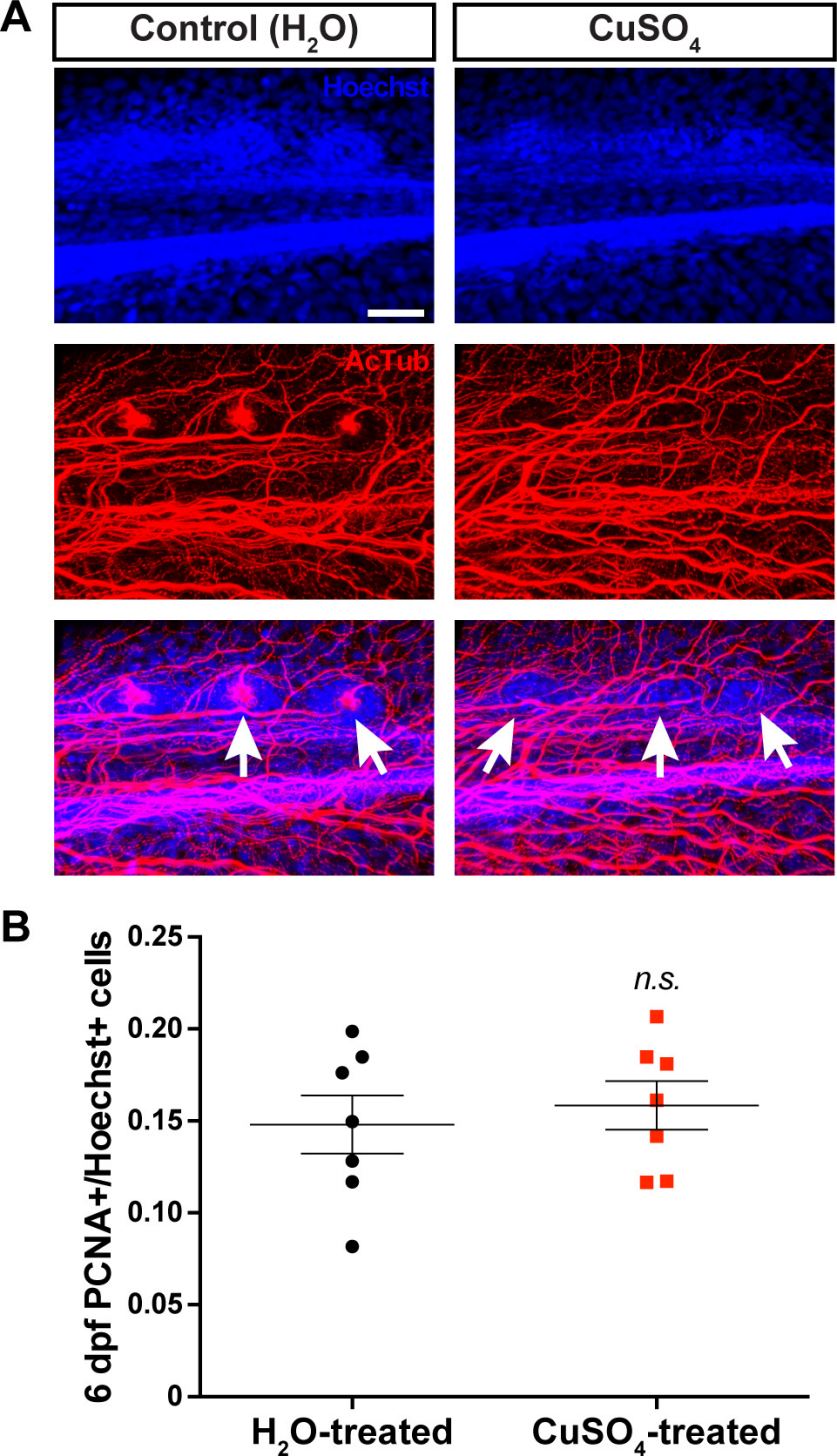
Ablation of the lateral line does not affect pallial cell proliferation. On 3 dpf, 30 min exposure to CuSO_4_ destroyed kinocilia (as visualized by acetylated tubulin) associated with hair cell cupulae (white arrows) along the larval zebrafish lateral line (**A**). Prior ablation of lateral line hair cells on 3 dpf did not affect the number of PCNA+ cells in the pallium reared in unrestrained conditions (B; n = 7). Scale bar = 40 µm. n.s., not significant. Data are represented as mean ± SEM.

One source of neural feedback that could detect physical movement is the lateral line, a system of hair cells distributed along the teleost body that detects changes in water flow (Dijkgraaf, 1963). We treated 3 dpf larvae with 30 µM copper sulfate for 30 min, an ototoxin that ablates lateral line hair cells and impedes subsequent regeneration of these cells over the following days (Mackenzie and Raible, 2012). We confirmed hair cell ablation by the complete absence of beta-acetylated tubulin (AcTub) expression in hair cell cuppulae following treatment with copper sulfate (Figure 5A). If the lateral line is involved in mediating movement-dependent neurogenesis, then removal of this feedback should affect PCNA+ cell populations in the pallium. However, copper sulfate treatment did not affect the proportion of PCNA+ cells in the 6 dpf larvae pallium (Figure 5B; ^31^t_12_ = 0.51, p=0.62) when all larvae were reared in unrestrained wells. Intact lateral line signaling does not appear to mediate movement-dependent changes in pallial neurogenesis.

### Larvae deficient in trunk dorsal root ganglia exhibit attenuated movement-dependent increases in pallial neuroproliferation

**Figure 6.**
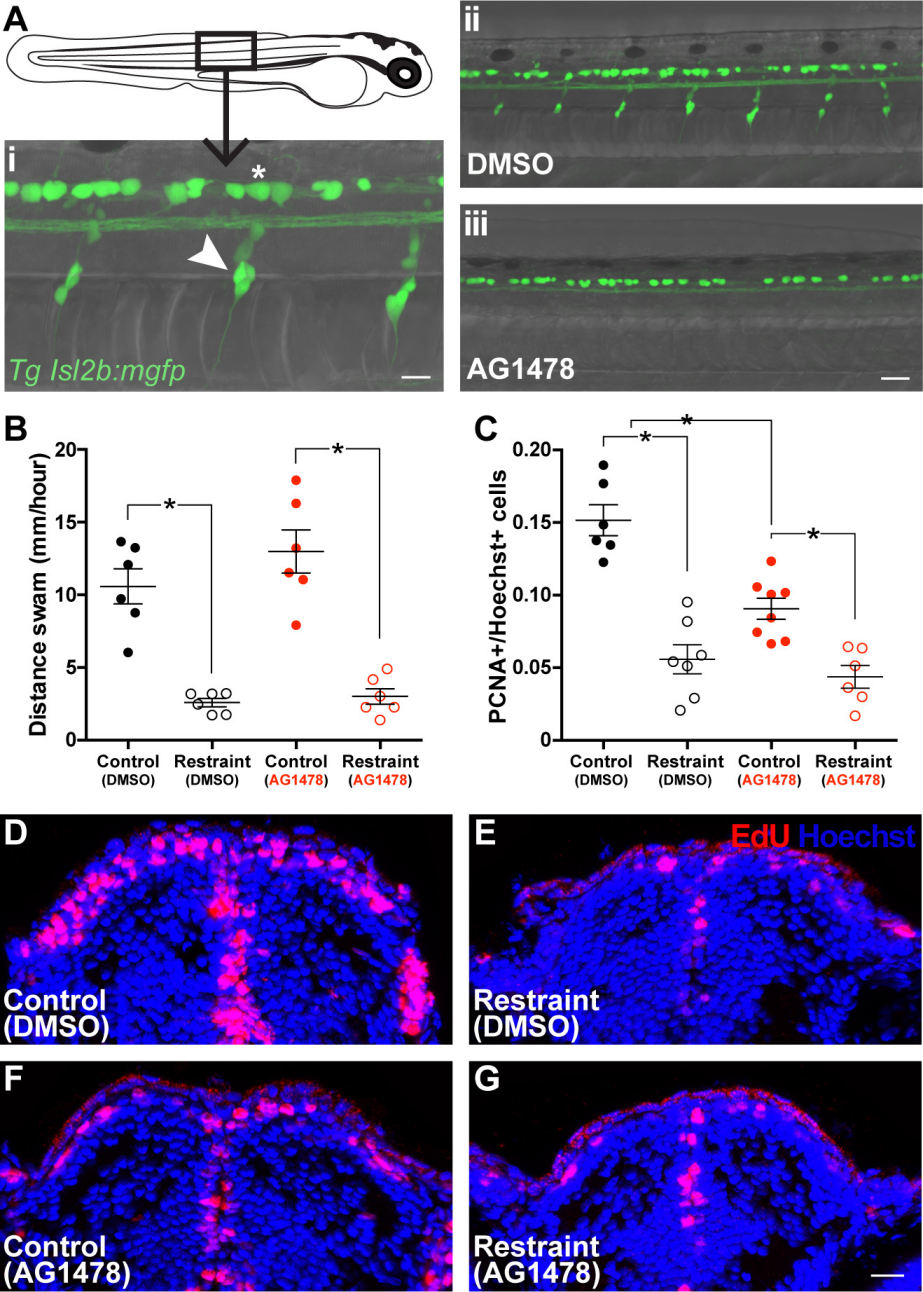
Impairing trunk DRG formation attenuates movement-dependent pallial neurogenesis. (Ai) Dorsal root ganglia (white arrow) and Rohon-Beard neurons (white asterisk) were visualized in *Tg(isl2b:mgfp)* larvae (scale bar = 40 µm). Treatment with AG1478 from 8 to 30 hpf prevented development of DRG along the trunk in larvae by 3 dpf without affecting RB neuron populations dorsal to the spinal cord (Aii-iii). Earlier treatment with AG1478 did not affect swimming compared to DMSO-treated controls on 8 dpf (B; n = 6). By 9 dpf, restrained larvae in both DMSO (F-G; control n = 6, restraint n = 7) and AG1478 (H-I; control n = 8, restraint n = 6) treatments exhibited fewer pallial PCNA+ cells compared to controls, however, AG1478-treated controls exhibited fewer pallial PCNA+ cells compared to DMSO-treated controls, despite similar swimming behaviour. *p<0.05. Data are represented as mean ± SEM.

In vertebrates, DRG collect sensory feedback from the body and communicate these signals via ascending pathways to the CNS in the spinal cord (Vandewauw et al., 2013). Accordingly, DRG represent another system of neural feedback that could convey physical cues associated with movement. We tested whether blocking DRG development in the trunk would reduce PCNA+ cell populations in the pallium associated with swimming. We blocked development of DRG along the larval trunk by treating embryos with the ErbB receptor antagonist AG1478 in a limited window from 8 to 30 hpf followed by a wash-out period of almost 2 days (Honjo et al., 2008). By 3 dpf, we confirmed that earlier AG1478 treatment reduced DRG development in *Tg(isl2b:mgfp)* transgenic embryos (Figure 6Ai–iii). However, AG1478 treatment affected neither the pallium size (Table 2) nor proportion of pallial PCNA+ cells on 3 dpf, prior to any motor treatments (Figure 6—figure supplement 1A; ^32^t_7_ = 0.04, p=0.97). Thus, we divided 3 dpf AG1478- and DMSO-treated larvae into control and movement restraint conditions and sampled PCNA+ cell populations as above.

**Figure 6—figure supplement 1.**
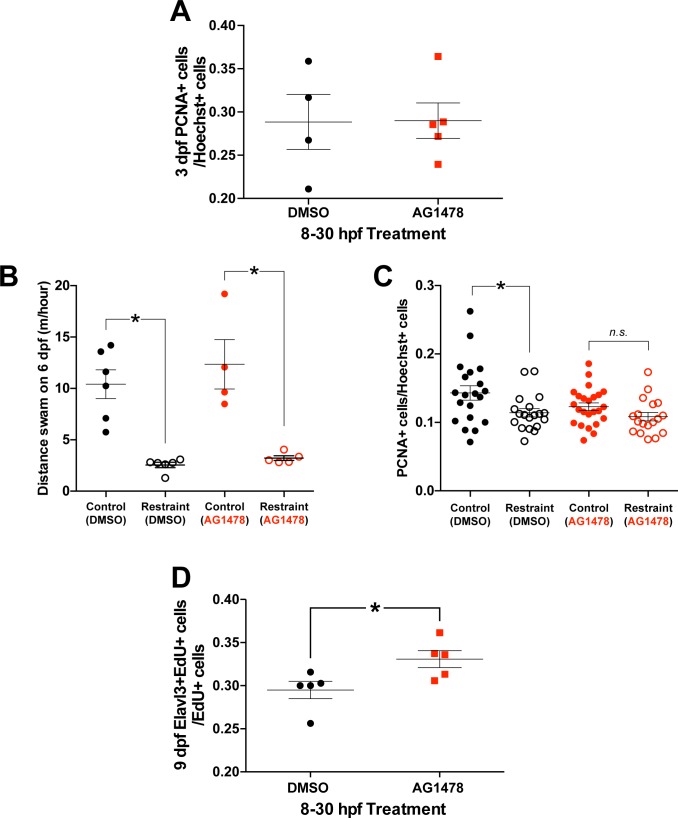
Treatment with AG1478 from 8 to 30 hpf did not affect the number of PCNA+ cells in the pallium by 3 dpf in zebrafish larvae (control n = 4, restraint n = 5). Treatment with AG1478 from 8 to 30 hpf did not affect swimming on 6 dpf in both restrained and unrestrained conditions compared to DMSO-treated controls (B; DMSO control n = 6, DMSO restraint n = 6, AG1478 Control n = 4, AG1478 Control n = 5). Treatment with AG1478 from 8 to 30 hpf eliminated the movement-dependent changes in the number of pallial PCNA+ cells by 6 dpf, whereas larvae treated with DMSO still exhibited movement-dependent neurogenesis (C; DMSO control n = 20, DMSO restraint n = 19, AG1478 Control n = 24, AG1478 Control n = 18). Treatment with AG1478 from 8 to 30 hpf increased Elavl3/EdU co-localization in unrestrained 6 dpf larvae exposed to EdU from 5 to 6 dpf (D; n = 5). *p<0.05. n.s. = not significant. Data are represented as mean ± SEM.

By 6 dpf, prior AG1478 treatment did not affect swimming compared to DMSO-treated controls (Figure 6—figure supplement 1B; ^33^F_3,17_ = 16.16, p<0.01). Whereas DMSO-treated larvae exhibited a movement-dependent change in the proportion of pallial PCNA+ cells, prior AG1478 treatment blocked this effect (Figure 6—figure supplement 1C; ^34^F_3,77_ = 4.17, p<0.01). Prior AG1478 treatment did not affect 6 dpf pallium size between unrestrained larvae (Table 2). Furthermore, when unrestrained larvae were exposed to EdU from 5 to 6 dpf as in our restraint paradigm, prior AG1478 treatment increased the number of newly generated (EdU+) cells that also express Elavl3 (Figure 6—figure supplement 1D; ^35^t_8_ = 2.53, p=0.04) compared to DMSO-treated controls, suggesting early AG1478 treatment affects pallial neurogenesis similarly to chronic restraint, albeit at a reduced magnitude. To resolve differences in the proportion of PCNA+ cells in the pallium of AG1478- and DMSO-treated larvae, we repeated this experiment and extended control and movement restraint conditions until 9 dpf as above.

Prior AG1478 treatment also did not affect swimming on 8 dpf (Figure 6B; ^36^F_3,20_ = 27.59, p<0.01). By 9 dpf, AG1478-treated larvae exhibited movement-dependent differences in the proportion of pallial PCNA+ cells, however, the magnitude of this effect was significantly attenuated compared to DMSO-treated controls (Figure 6C–G; ^37^F_3,23_ = 26.68, p<0.01). Prior AG1478 treatment did not affect 9 dpf pallium size between unrestrained larvae (Table 2). Together, these results suggest that neural feedback from DRG mediates, at least in part, movement-dependent forebrain neuroproliferation.

### Activating DRG in immobilized larvae is sufficient to increase pallial neuroproliferation

**Figure 7.**
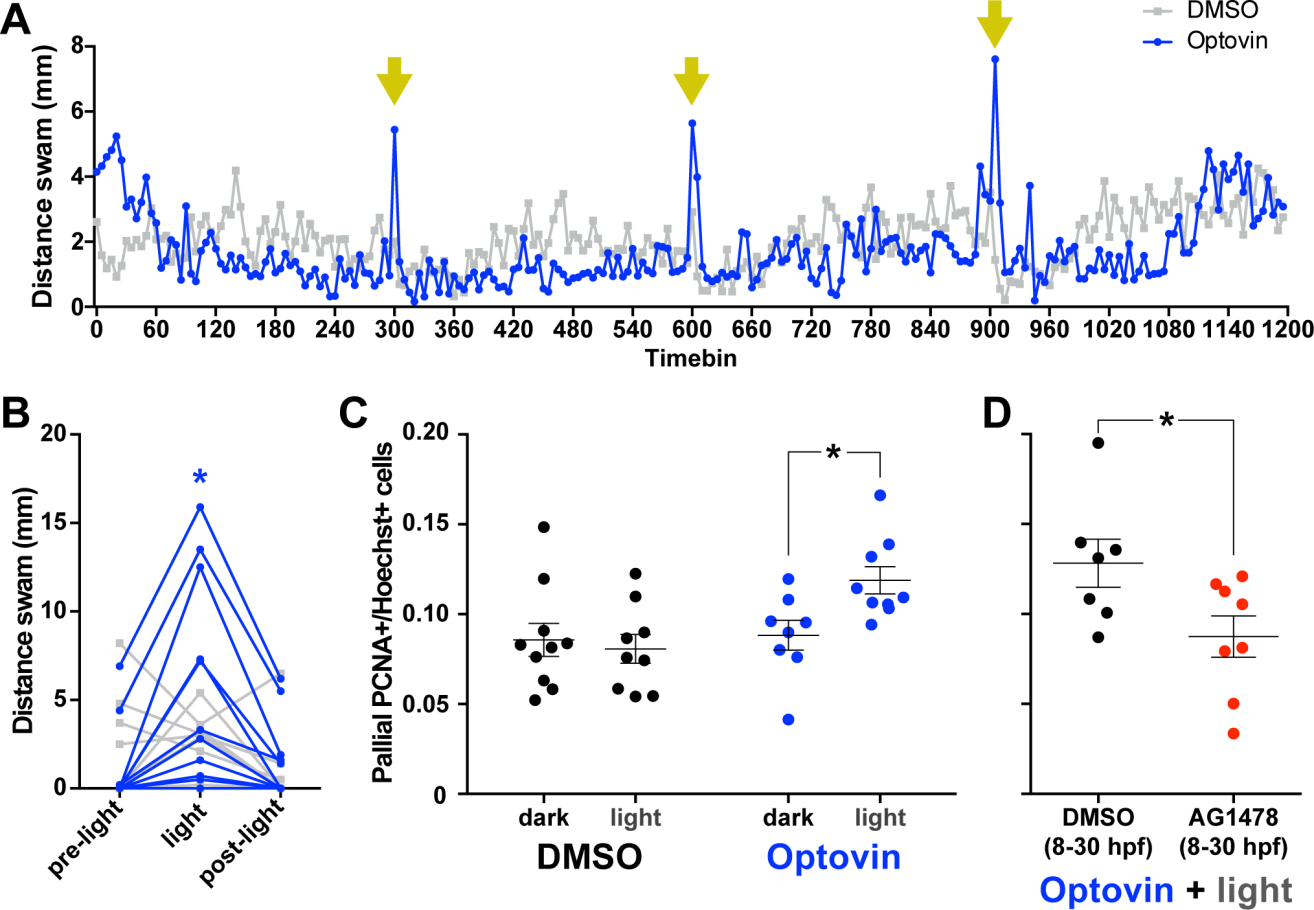
Photoactivation of DRGs using Optovin increases pallial neurogenesis. 5 dpf larvae incubated in Optovin exhibit repeatable movement bursts in response to light (**A**). Points on each line represent the mean distance swam (n = 12) during 5 s time bins by larvae treated with either Optovin (blue) or DMSO (grey) and exposed to 2 s of light (yellow arrows) every 5 min. (**B**) Expanded view of first light presentation from (**A**), including the 5 s of darkness prior to light presentation (pre-light), the 5 s time bin including light presentation for the first 2 s (light), and the following 5 s of darkness (post-light). Treating 5 dpf larvae immobilized in agarose with 2 s light pulses every 5 min for 5 hr increased the number of PCNA+ cells in the pallium 12 hr post treatment only when larvae were incubated with Optovin (C; DMSO dark n = 10, DMSO light n = 9, AG1478 dark n = 8, AG1478 light n = 9). Larvae treated with AG1478 from 8 to 30 hpf failed to exhibit an increase in the proportion of PCNA +cells in the pallium following this same Optovin + light treatment compared to controls (DMSO control n = 7, AG1478 n = 8). *p<0.05. Data are represented as mean ± SEM.

If DRGs mediate neural feedback during movement to stimulate pallial cell proliferation, then direct stimulation of DRGs independent of movement should also drive pallial neurogenesis. We used an optopharmacological approach to activate DRGs by exposing larvae to a combination of light and Optovin, a small molecule that enables photoactivation of TRPA1 receptors (Kokel et al., 2013). TRPA1 receptors are found in DRG (Vandewauw et al., 2013), trigeminal neurons and Rohon-Beard cells in larval zebrafish (Prober et al., 2008). In zebrafish, Optovin acts specifically on the TRPA1b paralog, which is exclusively expressed in sensory ganglia up to 5 dpf (Prober et al., 2008). To repeatedly photoactivate DRGs using Optovin, we exposed unrestrained 5 dpf larvae isolated in a 24-well plate to either Optovin or DMSO and adjusted light exposures and intermittent darkness to achieve repeatable behavioural activation. Unrestrained larval zebrafish incubated in Optovin exhibited intense, sporadic bouts of movement during light exposure, presumably as spinal reflexes in response to intense DRG activation (Kokel et al., 2013). 5 dpf larvae exhibited repeatable, photo-activated motor responses to 2 s of exposure to light every 5 min, whereas larvae treated with DMSO exhibited no such responses (Figure 7A–B, Figure 7—figure supplement 1; Treatment x Timebin Interaction: ^38^F_2,44_ = 6.36, p<0.01). Therefore, we used 2 s of light stimulation every 5 min as a paradigm to regularly stimulate DRG in immobilized larvae.

**Figure 7—figure supplement 1.**
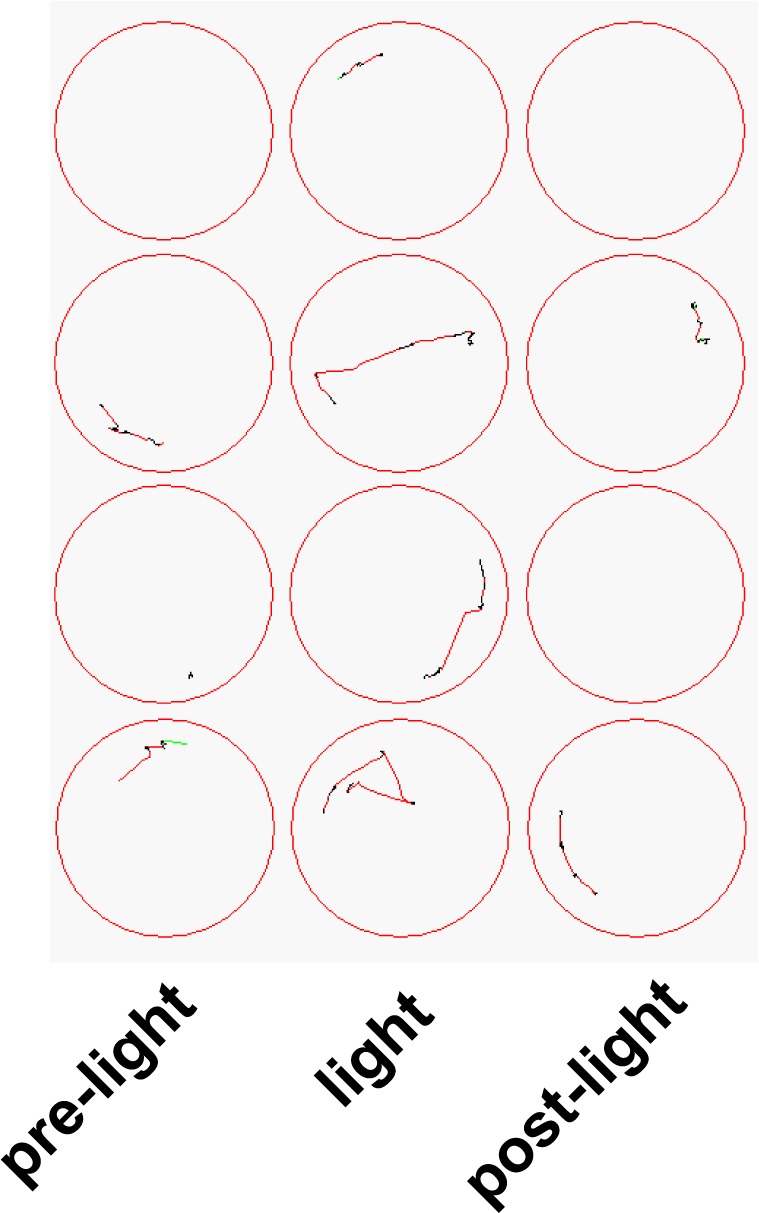
Example movement traces from 5 s timebins in 4 wells of larvae incubated with 10 µM Optovin prior to, during, and following a 2 s presentation of white light (800 lux).

We immobilized 3 dpf larvae in agarose individually in 24-well plates. On 5 dpf, larvae were incubated with either DMSO or Optovin and exposed to either darkness or a 5 hr session of light presentations as above. Twelve hours following the end of this session, light exposures significantly increased the proportion of PCNA+ cells in the pallium of larvae exposed to Optovin (Figure 7C; Drug x Light Treatment interaction; ^39^F_1,32_ = 4.47, p=0.04), whereas light treatments had no effect on the proportion of PCNA+ cells in DMSO-incubated larvae. Furthermore, 6 dpf larvae deficient in trunk DRGs (using a transient 8–30 hpf treatment with AG1478 as above) did not exhibit this optovin-and-light-dependent increase in the proportion of PCNA+ cells in the pallium compared to controls (8–30 hpf DMSO treatment; Figure 7D; ^40^t_13_ = 2.33, p=0.04). Thus, DRG activation appears sufficient to increase pallial neurogenesis in the zebrafish larvae in the absence of physical movement.

## Discussion

### Movement regulates postembryonic neurogenic forebrain growth in larval zebrafish by regulating progenitor cell differentiation

We found that movement plays a critical role in determining the number of neural progenitors in the zebrafish forebrain during postembryonic development. Previous work has focused on coupling increased physical activity via aerobic exercise with increases in cell proliferation in the adult mammalian SGZ (Fabel and Kempermann, 2008). Here, we found that physical activity also modulates forebrain cell proliferation postembryonically in the larval zebrafish pallium. Whereas we found that increased physical activity in fish led to an increase in pallial cell proliferation, we also report a negative neurogenic response when movement is reduced via restraint or immobilization. In the most extreme case, restricting larval movement resulted in the near absence of a proliferative population in the pallium by 9 dpf, even though these larvae were fully capable of swimming normally thereafter. Furthermore, we found that these changes in progenitor populations had subsequent impacts on neurogenic brain growth: restrained larvae, who exhibit reduced pallial cell proliferation by 6 dpf, develop smaller forebrains by 9 dpf due to a combination of reduced neurogenesis and, to a lesser extent, pallial cell apoptosis.

The mechanisms through which the neurogenic niche is affected by exercise in the adult rodent hippocampus include proposed changes in cell fate, cell cycling, and apoptosis in neural precursors (Overall et al., 2016). Here, we found that, postembryonically, movement appears to maintain proliferative cell populations in the zebrafish pallium primarily by promoting self-renewal in neural progenitor cell populations whereas restraining movement promoted their premature differentiation. Because control and restrained larvae produced the same number of cells in the forebrain from 5 to 6 dpf, movement-dependent regulation of postembryonic forebrain cell proliferation appears to occur predominantly through regulating self-renewal and the production of differentiated cells over factors that might affect the absolute number of cells produced, such as cell cycle length. Within the neurogenic niche, movement-dependent maintenance of the progenitor pool may involve the Shh signaling pathway, which expands progenitor populations via symmetric cell division (Lai et al., 2003; Machold et al., 2003; Yang et al., 2015). Collectively, our findings demonstrate the importance of movement in maintaining a source of new neurons to support forebrain growth postembryonically and present zebrafish as a novel model in which movement modulates early brain development over the course of a few days.

### Movement produces non-visual, non-lateral line neural feedback conveyed via DRG to affect pallial neurogenesis

In addition to characterizing the relationship between movement and postembryonic neurogenesis in the forebrain, we also sought to identify the nature of the feedback signal associated with movement that drives this neurogenic change. We found that physical cues associated with movement send ascending neural feedback to the brain via DRGs to drive changes in neurogenesis. Specifically, larvae deficient in DRGs along their trunk exhibited an attenuated neurogenic responses to swimming compared to controls. However, we still found significant modulation of pallial cell proliferation on 9 dpf in DRG-deficient larvae. This continued modulation of neurogenesis in the older larvae may be attributed to the nature of our treatment, which blocks the development of DRGs along the trunk, but does not affect DRG populations that are derived from neural crest cells in the head that may also signal movement (Honjo et al., 2008). Other proposed mechanisms of movement-dependent neurogenesis, such as the circulation of growth factors proposed to mediate exercise-dependent adult neurogenesis (Cotman et al., 2007), and other mechanosensory cell populations, such as Rohon-Beard cells, may also play a role in driving motor experience-dependent neurogenic brain development. A previous study has demonstrated that treating zebrafish embryos with AG1478 can impair proliferation during embryogenesis in the zebrafish optic tectum (Sato et al., 2015). In that study, changes in tectal neurogenesis were observed using a near 8-fold increase in AG1478 concentration (compared to that used here) and neurogenesis was found to resume normally within hours following drug washout. Recognizing the possibility of a lasting effect of early AG1478 treatment, we sampled forebrain neurogenesis in 3 dpf larvae treated earlier with AG1478 or DMSO and found no effect of AG1478 treatment on pallial neurogenesis prior to movement or Optovin manipulations. We also found that earlier AG1478 treatment had no effect of pallium size in restrained or unrestrained control larvae by 6 dpf. In conjunction with our original movement and Optovin experiments, which do not include AG1478 manipulations, our results suggest that motor experience-dependent neurogenesis is mediated, in part, via peripheral neural feedback and is likely not attributed to early AG1478 treatment. However, future work would benefit by contrasting the results obtained here with larvae in which DRG development is blocked using alternative means. Furthermore, the ErbB signaling inhibitor used in our studies may have non-neural effects on skin or heart development. Although it is unlikely that these could be a factor in determining transmission of movement information to the brain to alter forebrain neurogenesis, this could be examined in the future.

Using completely immobilized larvae, we were able to stimulate pallial cell proliferation by stimulating DRG along with other cells. Furthermore, this increase in pallial cell proliferation due to stimulation was not observed in larvae deficient in trunk DRGs. In conjunction with our studies reducing specific DRG populations along the trunk, our results suggest that neural feedback associated with movement is sensed predominantly by DRG and that DRG activation is sufficient to expand a progenitor pool in the forebrain. This previously undocumented role for DRG in conveying physical cues associated with movement to expand pools of forebrain progenitors may in turn provide a larger source of neurons and support more neurogenic brain growth in the most active animals.

### DRG are essential to receiving sensory input associated with movement independent of the lateral line

We found that physical movement of the body was the most important component of movement driving pallial neurogenesis. Accordingly, we propose that movement triggers mechanosensory input detected by DRGs that are then sent to the brain. In zebrafish larvae, mechanosensory input is most likely to come from one of three sources. The first possibility is the lateral line, which detects changes in water flow and vibration in the environment (Dijkgraaf, 1963). Here, ototoxic ablation of this system had no impact on pallial proliferation, suggesting it does not play a role in maintaining pallial progenitor populations. Second, Rohon-Beard (RB) cells, an early-developing population of spinal neurons, transmit mechanosensory signals (Faucherre et al., 2013) and contain TRPA1b receptors (Prober et al., 2008) that can be activated by Optovin stimulation (Kokel et al., 2013). Originally, RB cells were thought to die off entirely by 4 dpf (Reyes et al., 2004), but subsequent work using transgenic markers suggests they may persist up to 1–2 weeks post-fertilization (Kucenas et al., 2006; Palanca et al., 2013). Our studies showed that early AG1478 treatment, which specifically affected DRG development with resident RB cell populations remaining unaffected, blocked the Optovin-induced increase in proportional PCNA+ cells in the pallium following light presentations, suggesting DRG and not RB are responsible for the increase in pallial neurogenesis following Optovin and light treatment. However, our data indicate that RBs may also contribute to motor experience-dependent pallial cell proliferation, particularly in DRG-deficient larvae by 9 dpf. Finally, DRGs can transmit mechanosensory information from the trunk owing to the array of sensory channels they contain including TRPA1b (Kokel et al., 2013). Because both the removal and activation of DRGs produced neurogenic consequences in the pallium, we propose that these sensory neurons are the primary mediators of movement-dependent postembryonic neurogenesis.

TRPA1 channels exhibit deep evolutionary conservation across vertebrates (Christensen and Corey, 2007). Zebrafish have two orthologs of the TRPA1 channel: only TRPA1b, however, likely processes external signals (Prober et al., 2008) and is activated by Optovin treatment (Kokel et al., 2013). Whereas TRPA1 function has been implicated in touch stimuli in DRG of mice (Kwan et al., 2006; Brierley et al., 2011) and sensory neurons in *C. elegans* (Kindt et al., 2007), this channel is also associated with transducing chemosensory and nociceptive input (Prober et al., 2008). Here, we found evidence suggesting a novel function for TRPA1 in transducing physical cues associated with bodily movement during locomotion.

### Movement and neurogenic brain growth are coupled early in life

Our results demonstrate a robust connection between motor and brain development during postembryonic development. Motor development in most vertebrates begins early in the postembryonic period, including both viviparous species, such as with fetal motor development in humans (de Vries et al., 1982), and oviparous species, such as the larvae studied here. Therefore, if conserved across taxa, this close relationship between movement and neurogenesis may couple early motor and brain development. Furthermore, this relationship could help explain correlations between early physical and mental development, such as the long-observed comorbidity of physical and mental impairments (Barnett et al., 2012) and correlation between sedentary lifestyle and depression (Anton et al., 2006), which has been previously associated with impaired neurogenesis (Jacobs et al., 2000), in children.

## Materials and methods

**Key resources table:** 

Reagent type (species) or resource	Designation	Source or reference	Identifiers
Strain, strain background (*Danio rerio*)	Tg(dlx5/6:gfp)		ZFIN ID: ZDB-FISH-150901–22615
Strain, strain background (*D. rerio*)	Tg(GFAP:gfp)		ZFIN ID: ZDB-ALT-060623–4
Strain, strain background (*D. rerio*)	Tg(βactin:gfp)		ZFIN ID: ZDB-ALT-061107–2
Strain, strain background (*D. rerio*)	Tg(isl2b:gfp)		ZFIN ID: ZDB-FISH-150901–2212
Software, algorithm	Zebralab	ViewPoint, Montreal, Canada	
Software, algorithm	IMARIS	Bitplane, Belfast, United Kingdom	
Antibody	mouse anti-Human Neuronal Protein HuC/HuD	Life Technologies, Waltham, Massachusetts	A-21271; RRID: AB_221448
Antibody	mouse anti-PCNA	Invitrogen, Carlsbad, California	MA5-11358; RRID: AB_10982348
Antibody	rabbit anti-activated caspase 3	Cell Signaling Technology, Danvers, Massachusetts	Asp175; RRID: AB_2341188
Antibody	rabbit anti-GFP alexa 488-conjugated	Life Technologies, Waltham, Massachusetts	A-21311; RRID: AB_221477
Antibody	rabbit anti-tbr2	Abcam, Cambridge, United Kingdom	ab23345; RRID: AB_778267
Antibody	Cy3-conjugated goat anti-mouse	Jackson ImmunoReseach, West Grove, Pennsylvania	115-165-146; RRID: AB_2338690
Antibody	Cy3-conjugated goat anti-rabbit	Jackson ImmunoReseach, West Grove, Pennsylvania	111-165-003; RRID: AB_2338000
Antibody	Cy2-conjugated goat anti-rabbit	Jackson ImmunoReseach, West Grove, Pennsylvania	111-225-114
Chemical compound, drug	AG1478	Sigma-Aldrich	T4182 SIGMA
Chemical compound, drug	Optovin	Hit2Lead, San Diego, California	Chemibridge ID#5707191

### Animals

All zebrafish used in this study were of an AB genetic background. Larval strains used in this study include: *Tg(dlx5/6:gfp)* (generously provided by Dr. Marc Ekker, University of Ottawa) and *Tg(GFAP:gfp)* (generously provided by Dr. Pierre Drapeau, Université de Montreal) in motor restraint and visual vs. physical movement cue experiments; *Tg(βactin:gfp)* (generously provided by Dr. Ashley Bruce, University of Toronto) for copper sulfate treatments; and *Tg(isl2b:gfp)* (generously provided by the late Dr. Chi-Bin Chien, University of Utah) larvae for all AG1478 and Optovin treatment experiments. All adult zebrafish crossings included 2–3 male and female fish. Larvae were collected on the day of fertilization in system water and moved to a dark incubator held at 28°C. On 1 dpf, larval water was bleached for 30 s, rinsed four times with fresh system water, and larvae were dechorionated using forceps, before being returned to the incubator. From 3 dpf onward (dpf), larvae were housed in a facility room held under a 14/10 light/dark cycle at 28°C (Lights on at 08:00/Lights off at 22:00; light intensity = 300 lux). Larvae housed individually in well plates had half of their system water replaced twice daily (at 08:00 and 14:00). From 5 dpf onward, larvae were also fed size 0 zebrafish food (Gemma Micro; Skretting, Tooele, Utah) twice daily immediately following water changes. Zebralab (ViewPoint, Montreal, Canada) recordings were made in a separate testing room with similar environmental conditions as the fish facility. After recordings, all larvae were returned to the housing facility. In all experiments aside from those involving movement tracking (see below), larvae were randomly assigned to experimental conditions from the same clutch prior to experimental manipulations. Minimum sample sizes were selected to mirror those in preliminary experiments and our initial findings reported here, in which we found that restraint influenced the number of proliferative cells in the forebrain. Following all experimental procedures, larvae were sacrificed using an overdose of tricaine prior to tissue collection and fixation (see below). All animal experiments were performed with the approval of the University of Toronto Animal Care Committee in accordance with the guidelines from the Canadian Council for Animal Care (CCAC).

### Movement restraint apparatus

To restrict movement, we reared isolated 3 dpf larvae in wells of either unmodified 6-well plastic plates (well diameter = 3.5 cm) or modified 6-well plates in which larvae were confined to a central portion of each well within a cylinder of plastic mesh (Figure 1A; cylinder diameter = 1 cm). This mesh barrier was selected to both allow water flow in and out of the confined region and the rest of the well and prevent the larvae from escaping.

### Movement tracking

To track swimming of larvae throughout experiments, we used the Zebralab automated tracking system. Larvae were recorded on 4, 6, and 8 dpf. On each recording day, one tray of larvae was moved into the Zebrabox (ViewPoint; held at 800 lux light intensity) recording apparatus by 08:30. Following a 30 min habituation period, swimming was recorded for 4 hr before larvae were moved into fresh system water in a new tray and returned into the Zebrabox recording apparatus for a 30 min habituation and then an additional 4 hr of swimming before being returned to the facility room (at 17:00). On 6 and 8 dpf, larvae were also fed prior to habituation in the morning (at 08:00) and between recording sessions (at 13:00). Because only single trays of larvae were recorded in a session, restraint and control groups were reared as cohorts offset by 1 day and, accordingly, were recorded on alternating days. In all movement tracking experiments, we used 2 tanks of mixed-sex adult zebrafish (each containing 3 males and 3 females) from the same genetic background to generate each cohort. To control for genetic variation between families, parentage was reversed and balanced between treatment groups in subsequent cohorts. For example, the tank of adult zebrafish crossed to generate the control group in our first cohort was crossed to generate the restraint group in our second cohort. Tracking experiments were repeated to achieve the sample sizes reported here and included at least two cohorts. Zebralab tracking thresholds were set up as follows: Inactive/Small Swim Threshold = 5 mm/s; Small/Large Swim Threshold = 10 mm/s.

### Agarose embedding

To prevent physical movement from 3 to 6 dpf, we embedded larvae in 1.2% agarose dissolved in system water at 12:00 on 3 dpf. We moved larvae into plastic wells in a droplet of system water, anesthetized larvae using tricaine (4 g/L; Sigma-Aldrich, Oakville, Canada), and introduced a drop of 1.2% agarose to mix with the system water. Once set, additional warmed agarose was added around the embedded larvae to secure the embedded larvae in the well. For treatments requiring free tail movement, newly embedded larvae were submerged in system water and a scalpel was used to cut a block of agarose free from below the larvae’s neck to beyond the base of the tail.

### Visual grating

To simulate optic flow, we generated a visual grating stimulus using PsychoPy (Peirce, 2008) in which a black-and-white striped gradient moves along a randomly selected axis (between 0–360°) for 30 s, remains stationary for 30 s, and then begins again along a new, randomly selected axis. Gratings were displayed on a Dell P2212Hb computer monitor mounted horizontally, with plastic trays containing immobilized larvae sitting on top of the screen. In pilot experiments, grating bandwidth and speed were adjusted such that they would drive 6 dpf free-swimming larvae in a petri dish placed on the screen to swim along the grating axis. Gratings were presented to immobilized larvae starting on 3 dpf from 15:00-19:00, on 4–5 dpf for two 4 hr sessions (08:00-12:00 and 15:00-19:00), and on 6 dpf for just the morning session.

### Copper sulfate treatment

On 3 dpf, larvae were exposed to either system water or 30 µM copper sulfate (Sigma-Aldrich) added to the swimming media for 30 min starting at 09:00. Following copper sulfate exposure, both groups of larvae were rinsed three times with fresh system water. A subset of each treatment group was kept in a petri dish for an additional 30 min before sacrifice and tissue collection to validate ototoxicity of the copper treatment. The rest of the larvae were all isolated in control 6-well plates for the remainder of the experiment.

### AG1478 treatment

We dechorionated *Tg(isl2b:mgfp)* embryos by 6 hpf and treated them with either 4 µM 4-(3-chloroanilino)−6,7-dimethoxyquinazoline (AG1478), an ErbB receptor antagonist (Sigma-Aldrich) dissolved in 0.4% DMSO in system water or 0.4% DMSO in system water from 8 to 30 hpf (Honjo et al., 2008). At 30 hpf, larvae were rinsed three times with fresh system water and kept in an incubator until 3 dpf. On 3 dpf, AG1478 treatment was confirmed using fluorescence microscopy to count trunk DRG, which express GFP in *Tg(isl2b:mgfp),* in treated transgenic larvae. We excluded any larvae treated with AG1478 that exhibited more than 4 DRGs in the trunk. These larvae were removed from all experiments to ensure experimental manipulations only included larvae with most or all of trunk DRGs missing.

### Optovin treatment

We incubated larvae in Optovin (Hit2Lead, San Diego, California), a photo-activated small molecule that activates TRPA1b receptors found in zebrafish sensory neurons (Kokel et al., 2013). To establish a paradigm involving repeated photoactivation of TRPA1b, we incubated 5 dpf unrestrained larvae housed individually in 24 well plates in either 10 µM Optovin (in 0.1% DMSO in system water) or just 0.1% DMSO in system water for 2 hr in the Zebrabox (Viewpoint) tracking apparatus with the lights off. Following incubation, larvae were exposed to 2 s of white light (800 lux) alternating with 5 min of dark and movement was recorded using the automated tracking parameters outlined above.

After validating the utility of Optovin in unrestrained larvae, we incubated half of the larvae in 24-well plates containing 5 dpf larvae immobilized in agarose from 3 dpf (as above) in either Optovin or a DMSO vehicle. One tray of larvae was kept in the Zebrabox apparatus while the other was kept in complete darkness on the counter adjacent to the Zebrabox, beneath an opaque black plastic cover. From 15:00-20:00, larvae in the Zebrabox were exposed to 2 s of light (800 lux) alternating with 5 min of darkness. At 20:00, all larvae trays were rinsed using fresh system water three times and moved into the dark incubator overnight.

### Immunohistochemistry

For coronal section immunohistochemistry, larvae were sectioned using a freezing cryostat (20 μm sections), thaw-mounted on Superfrost Plus slides (Sigma-Aldrich), and dried for 3 hr in the dark at room temperature. Tissue was rehydrated in 0.2% Tween20 in phosphate-buffered saline (PBT) for 30 min at room temperature. At this point, tissue that was labeled for Elavl3 production was refixed with 4% paraformaldehyde for 20 min at room temperature and exposed to 50 mM Tris (pH = 8.0) for 60 min at 75–80°C before being rinsed with PBS three times and PBT once. All tissue was washed with PBT three times and blocked with 2% Normal Goat Serum (NGS) in PBT for at least 2 hr at room temperature. Tissue was incubated with the primary antibody in 2% NGS in PBT at 4°C overnight. Primary antibodies used in this study included mouse anti-Human Neuronal Protein HuC/HuD, also called ELAV like neuron-specific RNA binding protein three in zebrafish (Elavl3; Life Technologies, Waltham, Massachusetts, 1:400), PCNA (Invitrogen, Carlsbad, California, 1:500), rabbit anti-activated caspase 3, a marker of apoptosis (Cell Signaling Technology, Danvers, Massachusetts, 1:500), rabbit anti-GFP (Alexa-488 conjugated, Life Technologies, 1:1000), and tbr2 (Abcam, Cambridge, United Kingdom, 1:500). On the next day, tissue was rinsed three times with PBT before being incubated in a secondary antibody in 2% NGS in PBT for 1–2 hr at room temperature. Secondary antibodies used included Cy3-conjugated Goat Anti-Mouse IgG (Jackson ImmunoResearch, West Grove, Pennsylvania, 1:500), Cy3-conjugated Goat Anti-Rabbit IgG (Jackson ImmunoResearch, 1:500), and Cy2-conjugated Goad Anti-Rabbit IgG (Jackson ImmunoResearch, 111-225-144, 1:500). Tissue was rinsed with PBT three times. To visualize EdU, a Click-iT EdU reaction was performed as per the instructions included in the kit using the Alexa 647 azide (Invitrogen). Next, tissue was rinsed with PBT, counterstained with Hoechst for 10 min at room temperature, rinsed four times with PBS and coverslipped using 90% glycerol in PBS. Coverslips were sealed with clear nail polish and stored at 4°C until imaging. Images were captured with a Leica SP8 confocal microscope using a 40x objective as image stacks throughout the focus of sections compared as z-stacks with a z sampling distance of 1 μm. The treatment identity of larvae was masked prior to image analysis, which was all performed using IMARIS (Bitplane, Belfast, United Kingdom).

For whole mount immunohistochemistry larvae were fixed in 4% paraformaldehyde for 2 hr at room temperature, rinsed in PBS, exposed to acetone for 7 min at −20°C, rinsed again with PBS, and a mixture of 1% bovine serum albumin, 1% DMSO, and 0.1% TritonX-100 (PBDT) before being blocked in 10% NGS in PBDT for 1 hr. Following blocking, larvae were incubated overnight in mouse anti-alpha acetylated tubulin (Abcam, Cat No: 6-11B-1; 1:500). The second day of immunohistochemistry was completed as above, except exposure to the secondary antibody was extended to 5 hr at room temperature and whole mount larvae were kept in PBS at the end of staining, not coverslipped or sectioned.

### Cell counting

Images were captured with a Leica SP8 confocal microscope using a 40x objective as image stacks throughout the focus of sections compared as z-stacks with a z sampling distance of 1 μm. Image analyses performed using IMARIS (Bitplane, Belfast, United Kingdom). PCNA +cell sampling was performed using landmarks summarized in Figure 2—figure supplement 1. Olfactory bulb sampling was only possible in single, 20 µm sections and both hemispheres were sampled together. Both pallial and subpallial PCNA+, Hoechst+, tbr2+, and GFAP+ cell sampling were performed across both hemispheres in three consecutive, 20 µm sections for all histology involving 6 and 9 dpf larvae. PCNA +cell sampling in 3 dpf used only two consecutive coronal sections, as the telencephalon was not sufficiently grown to span 3 consecutive sections. Optic tectum sampling on 6 dpf was performed on the first coronal section posterior to the tectal neuropil, sampled in and averaged across both hemispheres in each brain.

### Statistical analysis

We define a biological replicate as an individual larvae derived from a mixed clutch borne of at least two male and two female adult zebrafish. Whereas we did not perform any technical replicates of our experiments, which we define as a complete repetition of a single experiment, we instead replicated our main findings by either repeating them in later, more elaborate experiments (for example, control vs. restraint paradigms in both initial experiments and AG1478 experiments) or repeating experiments over multiple time courses (for example, identifying the effects of exercise on forebrain neuroproliferation by both 6 and 9 dpf). All statistical test results are preceded by a superscript numeral enabling reference to each test in our calculations of statistical power summarized in Table 1. In experiments involving two groups, treatment groups were compared using Student’s t-test or, when parametric assumptions were not met, Mann-Whitney U tests. In experiments involving three or more groups, treatment groups were compared using either one-way ANOVA or two-way ANOVA (with a within-groups variable for repeated data). All post hoc comparisons were made using Tukey’s test with a correction for multiple comparisons. When neural precursor counts (PCNA+, tbr2+, and GFAP+ cells) were reanalyzed using the absolute number of cells/section (instead of corrected for the number of Hoechst+/cells), similar results were obtained as present here.

## Additional files

Source code 1. Python code used to generate moving grating.

Transparent reporting form
