# Supplementary material for: Movement maintains forebrain neurogenesis via peripheral neural feedback in larval zebrafish
Source: eLife. 2018 Mar 12;7:e31045. doi: 10.7554/eLife.31045 (PMC5847330; doi:10.7554/eLife.31045)
Supplement: Source code 1. [file elife-31045-code1.docx]

**Source Code File 1**

(Python code used to generate moving grating)

#create a window mywin = visual.Window([1920,1080],monitor="testMonitor", units="deg")

#stimuli grating = visual.GratingStim(win=mywin, mask='none', size=50, pos=[0,0], sf=.7) fixation = visual.GratingStim(mywin, size=0.2, pos=[0,0], color = 'black')

n = 240 # enable 4 hours run time

for i in range(n): . timer = core.CountdownTimer(60) #full iteration including moving grating for 30 and statio grating.ori = random.choice(range(0,360)) #random degree selection #draw the stimuli and update the window while timer.getTime() > 30: #30 second stimulus

grating.setPhase(0.085, '+')#advance grating cycle grating.draw() mywin.flip()

while timer.getTime() > 0 event.clearEvents()

lineo.draw() mywin.flip()
