# Supplementary material for: Movement maintains forebrain neurogenesis via peripheral neural feedback in larval zebrafish
Source: eLife. 2018 Mar 12;7:e31045. doi: 10.7554/eLife.31045 (PMC5847330; doi:10.7554/eLife.31045)
Supplement: Peer review history

# Decision letter

**DOI:** 10.7554/eLife.31045.022

**Affiliations:** Max Planck Institute for Heart and Lung ResearchGermany

In the interests of transparency, eLife includes the editorial decision letter and accompanying author responses. A lightly edited version of the letter sent to the authors after peer review is shown, indicating the most substantive concerns; minor comments are not usually included.

Thank you for submitting your article "Movement maintains forebrain neurogenesis via neural feedback through dorsal root ganglia in larval zebrafish" for consideration by *eLife*. Your article has been reviewed by three peer reviewers, and the evaluation has been overseen by Didier Stainier as the Senior Editor. The following individual involved in review of your submission has agreed to reveal her identity: Laure Bally-Cuif (Reviewer #1).

The reviewers have discussed the reviews with one another and the Senior Editor has drafted this decision to help you prepare a revised submission.

Summary:

The authors address here whether feedback relationships exist between physical activity and forebrain neurogenesis in the early zebrafish larva. This is a very interesting and unresolved question in the broad context of how sensory and physical modalities shape brain development in vertebrates. The authors combine behavior assays (affecting physical or vision-faked locomotion), pharmacological assays (affecting the lateral line, DRGs or RB neurons) and neurogenesis quantifications, to conclude that physical locomotion is positively correlated with pallial neurogenesis, and that this effect is in part mediated by DRG activity.

Overall, this is a carefully designed and interpreted piece of work, resulting in an interesting and novel finding. The manuscript at this stage lacks any direct insight into the exact circuitry and (neuro)mediators involved but is certainly the first to identify a link between locomotion and neurogenesis during stages of pallial construction.

Essential revisions:

1) The authors start by designing a restraint system that prevents fish from swimming to the extent that they normally do in a 3.5 cm well. Importantly, not only does restraint affect forebrain proliferation, but it also affects overall growth. The authors show that between 6 and 9 dpf, restrained larvae grow less than unrestrained larvae, but that despite this effect on growth, restrained larvae and unrestrained larva of the same age swim the same amount. My biggest concern about this manuscript is that the authors have not addressed the extent to which the overall growth deficit of restrained larvae contributes to their forebrain neural proliferation deficit. A possible way to examine this issue would be to compare fish of the same size, for example older restrained larvae that are the same body length as younger unrestrained larvae and see whether this contributes to differences in the number of progenitors and differentiating neurons. Alternatively, the authors could select individual restrained and unrestrained larvae of the same age and size for direct comparisons. They may already have the data in hand for this experiment if they tracked the body length of all of the larvae they studied, but that information isn't included in the manuscript.

Understanding the role of overall growth on the effect of movement on forebrain neurogenesis is also an important consideration elsewhere in the manuscript. For example, was overall growth affected in the experiments in which larvae experienced different levels of water flow? The authors sampled the number of Hoechst positive cells per section as a proxy for forebrain size and concluded that brain size wasn't affected. But they don't elaborate on how many sections they examined per brain and whether brains of larvae from the two conditions had the same number of brain sections.

2) For most of the behavioral or pharmacological assays used (except for the starting point of the paper, Figure 1 and Figure 2), the only read-out of "neurogenesis" is the number of PCNA-positive cells. The effects of restraining movements in a mesh are comprehensively analyzed by looking at proliferation (PCNA), cell types (Tbr2, GFAP), cell death (Cas3) and cell fate (EdU); however, PCNA is the sole measurement for all subsequent assays (in particular those serving as a control to movement restraint, such as excess swimming, Figure 3, or those assessing the role of DRGs, Figure 6—figure supplement 1, Figure 6 and Figure 7). Given that key interpretations appear conclusive only when opposite manipulations are considered jointly (notably re. the role of DRGs), I see it as important to better assess the neurogenesis phenotype in the different paradigms used. I would suggest, at least, to assess neural progenitor cell fate (using EdU chase) in complement to Figure 3, Figure 4 and Figure 6.

3) Several points were raised about AG1478 and Optovin:

– The authors found that blocking DRG formation with AG1478 had a significant effect at 6 dpf that diminished considerably by 9 dpf, it is worth considering what other cell types might be involved. The authors discuss the possibility of RB neurons, but don't perform any tests. As the authors report, RBs also express TRPA1b receptors, so perhaps the activation experiments affect forebrain neurogenesis via both DRGs and RBs. There may be subpopulations of RBs, which could suggest that some RBs are involved, along with DRGs, in movement-induced forebrain neurogenesis, or that DRGs and RBs act at different times in the process. Second, in addition to a requirement for ErbB signaling in DRG formation, ErbB signaling is also known to affect Schwann cell development and myelination, cardiac development, and skin development. Although it seems unlikely that these could be a factor in determining transmission of motility information to the brain to alter forebrain neurogenesis, without ruling them out it isn't certain, so at least they should be acknowledged.

– The ErbB antagonist AG1478 impairs mitosis, so the effects of the apparent DRG ablation on proliferation could in fact be a direct effect of the antagonist on the proliferative cells themselves (see PMID 26001123). The proper interpretation for this study is that decreasing proliferation of PCNA (+) cells does not alter swimming behavior. Moreover, trpa1 has been shown to be expressed in mammalian somatosensory context and so, making the assumption that expression patterns are conserved across fish and mammals, activation with Optovin might mediate effects within the CNS rather than the DRG (see PMC5413904). Given the off-target effects of these reagents, nothing can be said from these particular data about the effects of DRGs on CNS proliferation.

4) Sample sizes and origins of samples don't seem to be optimized in this study. Moreover, it seems from the methods that controls, and experimental fish came from separate sets of parents? Clarification on the number of crosses used, the number of individuals used from a given cross, and how the genetic background or family was controlled in experimental design or statistics should be provided. What was the genetic background of these fish (i.e., tinbergen, oregon, AB, etc.) and was it consistent across experimental groups and experiments?

5) Given that the authors' major focus is on locomotor activity (LMA), given that LMA is under circadian control, and given that neuroblast migration in zebrafish is under circadian control, more attention and clarity to zeitgebers and zeitgeber time should be provided. It seems that the authors acknowledge the possibility of circadian effects at least superficially when reporting some of the times of day that experiments were performed. However, time of day doesn't really matter. Rather time after lights on, intensity of light prior to and during LMA recording, feeding times (esp. important in zebrafish as a zeitgeber) etc., would be more valuable factors to readers and the authors for determining whether circadian effects including time of day and masking (esp. in the Optovin treatment experiment) have confounded the data. ZT and relevant CTs should be tested and ruled out as a contributing factor statistically.

6) Adding a table showing the brain regions where analyses were done will make it easier to follow and to understand which regions were surveyed, where things changed, and where things were unaffected. Along with the table, a clear description of the brain regions, how they were defined in these authors' hands, along with the information we all requested on possible changes in the sizes of these regions and numbers of cells would also help with interpretations and enable readers to understand the overall story.

[Editors' note: further revisions were requested prior to acceptance, as described below.]

Thank you for resubmitting your work entitled "Movement maintains forebrain neurogenesis via neural feedback through dorsal root ganglia in larval zebrafish" for further consideration at *eLife*. Your revised article has been favorably evaluated by Didier Stainier (Senior editor) and two reviewers, Laure Bally-Cuif and Chris Thompson.

The manuscript has been improved but there are some remaining issues that need to be addressed before acceptance, as outlined below:

Summary:

This manuscript describes experiments in zebrafish larvae testing the effects of experience on neurogenesis, differentiation, and survival in the developing nervous system. The authors test this using a variety of techniques, including environmental modifications to alter behavior and pharmacological manipulation of sensory input and neural activity, and quantify changes in proliferation using a well-characterized antibody (PCNA). They also use other antibodies to assess differentiation and cell death, and also assess new cell survival using the thymidine analogue EdU. They conclude that diminished swimming behavior reduces neural proliferation but increases over differentiation, and that this effect is dependent upon normal activity in the DRG.

The authors have conducted very thorough and detailed revisions and bring in a considerable amount of new information to confirm the solidity of their methodologies or interpretations. This is a highly interesting and convincing piece of work.

Please consider the following 3 points:

1) Given the results from (PMID 26001123), which show very clear effects of AG1479 on proliferation in the developing zebrafish brain, the authors should compare their results to this paper in the Discussion section.

2) Along the same lines, the title may too strongly state that the phenomenon is mediated via the DRG. Given the caveats of AG1479, some revision of the title might be warranted, and so a more "neutral" title, simply mentioning the link between movement and neurogenesis at larval stages, might be appropriate.

3) the manuscript would benefit by providing access to videos of the restricted swimming behavior paradigm, as it would help readers to conceptualize the impact of the mesh barrier on the swimming behavior.

# Reply: Author response

**DOI:** 10.7554/eLife.31045.023

Summary:

The authors address here whether feedback relationships exist between physical activity and forebrain neurogenesis in the early zebrafish larva. This is a very interesting and unresolved question in the broad context of how sensory and physical modalities shape brain development in vertebrates. The authors combine behavior assays (affecting physical or vision-faked locomotion), pharmacological assays (affecting the lateral line, DRGs or RB neurons) and neurogenesis quantifications, to conclude that physical locomotion is positively correlated with pallial neurogenesis, and that this effect is in part mediated by DRG activity.

Overall, this is a carefully designed and interpreted piece of work, resulting in an interesting and novel finding. The manuscript at this stage lacks any direct insight into the exact circuitry and (neuro)mediators involved but is certainly the first to identify a link between locomotion and neurogenesis during stages of pallial construction.

We are grateful for the positive feedback and appreciate the reviewer’s acknowledgement of the novelty of our findings.

Essential revisions:

1) The authors start by designing a restraint system that prevents fish from swimming to the extent that they normally do in a 3.5 cm well. Importantly, not only does restraint affect forebrain proliferation, but it also affects overall growth. The authors show that between 6 and 9 dpf, restrained larvae grow less than unrestrained larvae, but that despite this effect on growth, restrained larvae and unrestrained larva of the same age swim the same amount. My biggest concern about this manuscript is that the authors have not addressed the extent to which the overall growth deficit of restrained larvae contributes to their forebrain neural proliferation deficit.

We understand the concerns of the reviewers regarding the role of overall growth in the relationship between movement and forebrain neurogenesis. In the manuscript we provide a two-pronged approach demonstrating that body length can be dissociated from changes in the number of pallial PCNA^+^ cells using paradigms in which movement is both restrained and increased.

1) First, we would like to reiterate data already present in the original manuscript demonstrating that changes in forebrain neurogenesis occur between groups of fish that are the same size but have different movement experiences. In our restraint studies, 6 dpf represents a time point in which body length is not different (Figure 1—figure supplement 1A) between restrained and control larvae, but the number of forebrain PCNA^+^ cells is disproportionately lower in restrained larvae (Figure 2A; Figure 1—figure supplement 2A, B). This disproportionate decrease in the number of forebrain PCNA^+^ cells is also notable in that no such differences were detected in these same treatment groups in either the olfactory bulb or optic tectum (Figure 2—figure supplement 2C, D). Furthermore, we present new data summarized in Table 2 demonstrating that pallium and subpallium size did not differ on 6 dpf between control and restrained larvae. We concluded that by 6 dpf, movement experience could influence progenitor cell proliferation in the pallium without affecting overall growth of the brain or body. Nonetheless, this change in neuroproliferation in restrained larvae should ultimately have some consequence on brain growth. Thus, we opted to continue restraint until 9 dpf to characterize the developmental consequences of chronically reduced movement. Accordingly, we observed a decrease in forebrain size and this was also associated with an overall decrease in growth (body length). Thus, the forebrain neural proliferation deficit in restrained larvae precedes any overt changes in brain growth or body growth, which are only evident under relatively chronic (9 dpf) restraint conditions.

2) Second, we introduce new data demonstrating that when larvae were reared against strong water current, changes in forebrain neurogenesis could not be explained by changes in overall growth. We have incorporated this data into the manuscript to show that body length is not affected by fast water current rearing by 9 dpf (Figure 3G), whereas the number of proportional PCNA^+^ cells in the pallium is significantly higher in larvae reared in a fast current compared to controls (Figure 3D). Furthermore, we show that fast water current rearing did not affect pallium size by 9 dpf (Table 2), consistent with the notion that motor experience could affect neuroproliferation specifically without affecting overall growth. Along with 6 dpf larvae in our restraint paradigm, these two experiments demonstrate that movement-related changes in pallial neurogenesis cannot be explained by changes in overall growth.

A possible way to examine this issue would be to compare fish of the same size, for example older restrained larvae that are the same body length as younger unrestrained larvae and see whether this contributes to differences in the number of progenitors and differentiating neurons. Alternatively, the authors could select individual restrained and unrestrained larvae of the same age and size for direct comparisons. They may already have the data in hand for this experiment if they tracked the body length of all of the larvae they studied, but that information isn't included in the manuscript.

The first comparison proposed by the reviewer is interesting. However, we believe that comparing larvae of different age groups is complicated by potential age-related changes in neurogenesis that have been observed in the zebrafish brain later in life (e.g., PMID 23641971). Alternatively, the reviewer suggested we compare larvae of the same age and size for differences in forebrain neurogenesis. In our response to the comments above, we outline how this approach was the solution already taken in our restraint paradigm and is also supported by our newly added data on body length in fast water current-reared larvae as well.

Understanding the role of overall growth on the effect of movement on forebrain neurogenesis is also an important consideration elsewhere in the manuscript. For example, was overall growth affected in the experiments in which larvae experienced different levels of water flow? The authors sampled the number of Hoechst positive cells per section as a proxy for forebrain size and concluded that brain size wasn't affected. But they don't elaborate on how many sections they examined per brain and whether brains of larvae from the two conditions had the same number of brain sections.

We addressed the question of whether overall growth was affected in the fast water current experiments above. To clarify our sampling paradigms used for all PCNA measurements, we have included both Figure 2—figure supplement 1, which depicts how brain regions were sampled in coronal micrographs, and a “Cell counting” subsection in our Methods section. Important to this specific comment, all 6 and 9 dpf larval forebrains were sampled across three consecutive coronal sections per brain in every treatment group. We apologize for this omission.

2) For most of the behavioral or pharmacological assays used (except for the starting point of the paper, Figure 1 and Figure 2), the only read-out of "neurogenesis" is the number of PCNA-positive cells. The effects of restraining movements in a mesh are comprehensively analyzed by looking at proliferation (PCNA), cell types (Tbr2, GFAP), cell death (Cas3) and cell fate (EdU); however, PCNA is the sole measurement for all subsequent assays (in particular those serving as a control to movement restraint, such as excess swimming, Figure 3, or those assessing the role of DRGs, Figure 6—figure supplement 1, Figure 6 and Figure 7). Given that key interpretations appear conclusive only when opposite manipulations are considered jointly (notably re. the role of DRGs), I see it as important to better assess the neurogenesis phenotype in the different paradigms used. I would suggest, at least, to assess neural progenitor cell fate (using EdU chase) in complement to Figure 3, Figure 4 and Figure 6.

We thank the reviewer for this insightful comment and agree that demonstrating whether forebrain neurogenesis is modulated similarly across our experiments is critical to understanding the impact of motor experience on postembryonic pallial development. As suggested by the reviewer, we have repeated our Elavl3/EdU co-labeling experiment for the two most relevant experiments complementing our restraint paradigm (Figure 2M, N), in larvae reared against a fast water current, and in larvae deficient in trunk DRGs. In our strong current-rearing experiment, we were unable to expose larvae to a complete 24 hours of EdU prior to sacrifice because fresh system water constantly passes the larvae during swimming sessions. Instead, after the second swim on 8 dpf, each treatment group was transported into a separate petri dish and exposed to 5 mM EdU for 13 hours overnight. Following EdU exposure, now on 9 dpf, larvae were transferred back into their swimming canals for a final 5 hour swim and then sacrificed. We found that larvae reared in a strong current exhibited a decrease in the number of EdU+Elavl3+ co-labeled cells (Figure 3F), consistent with increased movement prioritizing the production of proliferative cell populations over differentiated neurons in the pallium. We also repeated the same EdU chase paradigm on unrestrained AG1478- and DMSO-treated larvae, to test whether a deficiency in trunk DRGs biases forebrain neurogenesis to produce more differentiated cells as seen following movement restraint. We found that 6 dpf AG1478-treated larvae exhibit an increase in the number of Elavl3/EdU co-labeled cells compared to DMSO-treated controls (Figure 6—figure supplement 1D), similar to the effects of movement restraint. In both of these experiments, we noted that group differences in EdU/Elavl3 co-labeling are attenuated compared to those in our restraint experiment, which is consistent with a similar difference in magnitude reported in our PCNA labeling experiments when comparing similar treatment groups.

The reviewer also suggested incorporating this EdU pulse-chase paradigm into the experiment presented in Figure 4. However, because our tail movement vs. visual stimulation experiment included larvae that are differentially exposed to agarose (tail cut free compared to fully embedded), we believe this may interfere with the exposure of EdU dissolved in the system water differentially between groups potentially leading to technical complications in the labeling. Given this limitation, we feel that the new data described above adequately addresses the reviewer’s comment.

3) Several points were raised about AG1478 and Optovin:

– The authors found that blocking DRG formation with AG1478 had a significant effect at 6 dpf that diminished considerably by 9 dpf, it is worth considering what other cell types might be involved. The authors discuss the possibility of RB neurons, but don't perform any tests. As the authors report, RBs also express TRPA1b receptors, so perhaps the activation experiments affect forebrain neurogenesis via both DRGs and RBs. There may be subpopulations of RBs, which could suggest that some RBs are involved, along with DRGs, in movement-induced forebrain neurogenesis, or that DRGs and RBs act at different times in the process. Second, in addition to a requirement for ErbB signaling in DRG formation, ErbB signaling is also known to affect Schwann cell development and myelination, cardiac development, and skin development. Although it seems unlikely that these could be a factor in determining transmission of motility information to the brain to alter forebrain neurogenesis, without ruling them out it isn't certain, so at least they should be acknowledged.

First, we would like to clarify that our AG1478 experiments do not necessarily show a significant effect on 6 dpf that is diminished by 9 dpf. On 6 dpf, we found that DMSO-treated larvae exhibit a significant difference in the number of forebrain PCNA^+^ cells between Control and Restraint groups, whereas no such difference was detectable in AG1478-treated larvae (Figure 6—figure supplement 1C). However, we were unable to resolve whether the number of pallial PCNA^+^ cells differed between unrestrained larvae in the DMSO- and AG1478-treated groups. We believe clarifying this distinction was critical towards understanding the importance of DRG in movement-dependent pallial neurogenesis. To resolve this difference, we repeated the experiment and extended all treatments until 9 dpf. On 9 dpf, we found that unrestrained AG1478-treated larvae exhibit significantly less forebrain PCNA^+^ cells than unrestrained DMSO-treated larvae (Figure 6C), suggesting DRG are at least partially involved in movement-dependent pallial neurogenesis. However, on 9 dpf we also found that AG1478-treated larvae still exhibit a degree of movement-dependent neurogenesis (Figure 6C). Together, these findings suggest that trunk DRG play a role in mediating movement-dependent pallial neurogenesis, but that other mechanisms are also involved.

As mentioned by the reviewer, our Optovin treatment could possibly be mediated by RBs, which express TRPA1b receptors. This is an excellent point that needed to be addressed experimentally. Accordingly, we present new data in which we tested whether early treatment with AG1478 (to generate trunk DRG-deficient larvae that still have the normal number of RB cells) would block the Optovin-and-light-dependent increase in the number of pallial PCNA^+^ cells from 5-6 dpf. All larvae were embedded in agarose on 3 dpf and, on 5 dpf, incubated with Optovin and exposed to the same light treatment paradigm as previously reported (both treatment groups together in the same Zebrabox at the same time). We found that AG1478-treated larvae failed to exhibit the increase in the number of pallial PCNA^+^ cells following light-and-Optovin treatment compared to DMSO-treated larvae, with means of both groups consistent with those observed in our previous Optovin experiment (Figure 7D). This finding suggests that DRG specifically mediate the Optovin-dependent increase in pallial PCNA from 5-6 dpf.

Together, these findings suggest that DRG play an important role in mediating movement-dependent pallial neurogenesis; however, other systems are likely involved. We have expounded on these points in the relevant Discussion section (subsection “Movement produces non-visual, non-lateral line neural feedback conveyed via DRG to affect pallial neurogenesis”; subsection “DRG are essential to receiving sensory input associated with movement independent of the lateral line”), including proposing a role for RB that may also contribute to a persistent difference in the number of pallial PCNA^+^ cells between restrained and control 9 dpf larvae, even following AG1478 treatment.

Finally, the reviewer indicates the possibility of non-DRG specific effects of the inhibition of ErbB signalling. We agree with the reviewer that changes in skin, myelination, or cardiac development are less parsimonious possibilities for explaining the transmission of movement information to the brain to modulate neurogenesis. Nonetheless, as requested by the reviewer, we acknowledged these less likely possibilities for future consideration in the Discussion section (subsection “Movement produces non-visual, non-lateral line neural feedback conveyed via DRG to affect pallial neurogenesis”).

– The ErbB antagonist AG1478 impairs mitosis, so the effects of the apparent DRG ablation on proliferation could in fact be a direct effect of the antagonist on the proliferative cells themselves (see PMID 26001123). The proper interpretation for this study is that decreasing proliferation of PCNA (+) cells does not alter swimming behavior. Moreover, trpa1 has been shown to be expressed in mammalian somatosensory context and so, making the assumption that expression patterns are conserved across fish and mammals, activation with Optovin might mediate effects within the CNS rather than the DRG (see PMC5413904). Given the off-target effects of these reagents, nothing can be said from these particular data about the effects of DRGs on CNS proliferation.

With all due respect to the reviewer’s point that “…nothing can be said from these particular data about the effects of DRGs on CNS proliferation”, we believe there are several convincing reasons why a direct effect of the AG1478 on pallial progenitor cells does not explain our data.

1) We are aware that the study referenced by the reviewer (PMID 26001123) reported a direct effect of the AG1478 inhibitor on neuroproliferation in zebrafish embryos. However, there are significant differences in the methodologies of this study compared to those used in our manuscript. To mention one example, these authors used 30 µM of AG1478, which represents a 8-fold increase in concentration compared to our use of 4 µM. They also used a 2% DMSO solvent concentration for the drug and control treatment group, whereas we do not go above 0.4% DMSO. In our hands, 2% DMSO is highly toxic to embryos. Importantly, while these authors treated embryos from ~1 dpf to ~2 dpf and reported changes in proliferation in the optic tectum (not the forebrain), they also clearly showed that after the drug was washed out, the proliferation defects quickly normalized within hours of the washout period and was therefore completely reversible almost immediately. We would like to emphasize that every AG1478 treatment reported in our manuscript only involved exposing embryos to this drug at a much lower concentration from 8-30 hours post-fertilization (hpf), after which the drug was rinsed from the embryo media and larvae were never exposed to AG1478 again. In fact, we only start treating larvae in movement restraint or strong water currents at 3 dpf, which is almost 2 days after the washout of the AG1478 drug.

Previously aware of this finding in their paper, we tested whether 8-30 hpf treatment with the AG1478 would have lasting effects on forebrain structure or neurogenesis in larvae at 3 dpf, prior to any movement manipulations, in our original manuscript. We found no effect of earlier drug treatments on the number of PCNA^+^ cells in the 3 dpf forebrain (Figure 6—figure supplement 1A). Furthermore, we include new data in the revised manuscript demonstrating that this early AG1478 treatment did not affect pallium size on 3 or 6 dpf (Table 2). Accordingly, we disagree with the reviewer that our experiment was primarily a manipulation of mitosis followed by a swimming assay. Instead, our study shows that the AG1478 treatment effectively generates trunk DRG deficient larvae (as originally demonstrated by Cotman, Berchtold and Christie, (2007)) without impacting pallial neurogenesis or pallium growth directly prior to postembryonic movement manipulations.

2) Regarding the potential central expression of TRPA1 in zebrafish larvae, unlike mammals, zebrafish contain two paralogs of the TRPA1 gene, TRPA1a and TRPA1b. These paralogs exhibit largely segregated expression profiles. As mentioned in the manuscript, TRPA1b is the paralog upon which Optovin acts. Prober et al., (2008, Figure 1G) found that TRPA1b is exclusively expressed in sensory ganglia and exhibits no central brain expression up to at least 5 dpf, the age at which we treated larvae with Optovin and light. We have added this detail to the manuscript (subsection “Activating DRG in immobilized larvae is sufficient to increase pallial neuroproliferation”). Consistent with the importance of TRPA1b in sensory ganglia mediating the Optovin response, Kokel et al., (2013, Figure 5) found that the Optovin-and-light-dependent motor response could be triggered in a spinalized adult zebrafish. Finally, as mentioned above, we present new data demonstrating the Optovin-dependent increase in the number of pallial PCNA^+^ cells from 5-6 dpf can be blocked by prior AG1478 treatment (Figure 7D), suggesting DRG play a critical role in mediating this neurogenic response.

4) Sample sizes and origins of samples don't seem to be optimized in this study. Moreover, it seems from the methods that controls, and experimental fish came from separate sets of parents? Clarification on the number of crosses used, the number of individuals used from a given cross, and how the genetic background or family was controlled in experimental design or statistics should be provided. What was the genetic background of these fish (i.e., tinbergen, oregon, AB, etc.) and was it consistent across experimental groups and experiments?

As outlined in our original manuscript, sample sizes were selected based on preliminary experiments and the initial studies performed identifying a restraint-dependent reduction in forebrain PCNA (subsection “Animals”).

We apologize for confusion regarding origins of samples in our experiments. First, all zebrafish used in this study were of an AB genetic background and we have added this information as the first sentence under subsection “Animals” in our Materials and methods section. To further clarify, the only experiments in which controls and experimental fish did not come from the same clutch were in experiments in which Zebralab was used to track locomotor activity on 4, 6, and 8 dpf. As we recorded swimming behaviour for 8 hours each testing day, we were only able to record one group each day. In hopes of minimizing variation, we divided adult fish into two mixed-sex tanks. In our first cohort of recordings, we collected a control clutch from crossing the first tank of adults (“Tank A”) and a restraint clutch from crossing the second tank of adults (“Tank B”) the following day. As we repeated restraint experiments to obtain additional cohorts, the parentage was reversed and balanced. For example, our second cohort consisted of a restraint clutch born from crossing “Tank A” (first used for control larvae) and a control clutch born from crossing “Tank B” (first used for restraint larvae). We have clarified this detail in our Materials and methods section (subsection “Animals” and subsection “Movement tracking”).

Importantly, we also clarify that larvae reared for Zebralab experiments were not used for histological analysis of forebrain neurogenesis. All non-Zebralab experiments included clutches born from crossing 2-3 adult males and females from the same genetic background and randomly assigning embryos/larvae from the same clutch to treatment groups. We have clarified this random assignment procedure in our Materials and methods section(subsection “Animals”). Thus, in response to the reviewer’s concerns, we have controlled for genetic and familial background of our fish in Zebralab experiments by using at least 2 clutches to generate data in which parentage was reversed and balanced across clutches and by randomly assigning larvae from the same clutch in all other experiments.

5) Given that the authors' major focus is on locomotor activity (LMA), given that LMA is under circadian control, and given that neuroblast migration in zebrafish is under circadian control, more attention and clarity to zeitgebers and zeitgeber time should be provided. It seems that the authors acknowledge the possibility of circadian effects at least superficially when reporting some of the times of day that experiments were performed. However, time of day doesn't really matter. Rather time after lights on, intensity of light prior to and during LMA recording, feeding times (esp. important in zebrafish as a zeitgeber) etc., would be more valuable factors to readers and the authors for determining whether circadian effects including time of day and masking (esp. in the Optovin treatment experiment) have confounded the data. ZT and relevant CTs should be tested and ruled out as a contributing factor statistically.

As requested, we have added the times of lights on/lights off in our Materials and methods section to contextualize time of day relative to our light scheduling, feeding times, and intensity of light in home rooms and our Zebralab recording apparatus (subsection “Animals”, subsection “Movement restraint apparatus” and subsection “Movement tracking”).

When designing our experiments, we aimed at avoiding issues regarding circadian rhythmicity by rearing control and experimental groups together or following the same daily schedule. As clarified above, outside of movement tracking experiments, we reared all treatment groups simultaneously, feeding them at the same time, exposing them to the same environmental conditions in the facility room, and importantly sacrificing all treatment groups simultaneously in each experiment. In movement tracking experiments, we reared cohorts following the same daily schedule, including timed feeds and movements between the Zebralab recording room and facility room. In our Optovin experiment, we exposed some treatment groups to pulses of light not experienced by dim groups. Aware of the potential differences in circadian entrainment attributed to lighting, we also ran a DMSO+light group, which did not exhibit an upregulation in the number of pallial PCNA^+^ cells (Figure 7C), suggesting lighting alone does not account for changes in Optovin+light-induced pallial neurogenesis. Accordingly, we are unable to identify environmental cues differentially experienced between treatment groups that would override the group differences reported here.

6) Adding a table showing the brain regions where analyses were done will make it easier to follow and to understand which regions were surveyed, where things changed, and where things were unaffected. Along with the table, a clear description of the brain regions, how they were defined in these authors' hands, along with the information we all requested on possible changes in the sizes of these regions and numbers of cells would also help with interpretations and enable readers to understand the overall story.

We agree that this information would greatly benefit the reader. As mentioned above, we have included Figure 2—figure supplement 1, in which our sampling paradigms used in coronal micrographs are depicted. We have also included Table 2, which summarizes brain region sampling and includes new analyses in which pallium size (Hoechst+ cells /section) was compared across additional experiments using the same datasets analyzed for PCNA.

[Editors' note: further revisions were requested prior to acceptance, as described below.]

Summary:

This manuscript describes experiments in zebrafish larvae testing the effects of experience on neurogenesis, differentiation, and survival in the developing nervous system. The authors test this using a variety of techniques, including environmental modifications to alter behavior and pharmacological manipulation of sensory input and neural activity, and quantify changes in proliferation using a well-characterized antibody (PCNA). They also use other antibodies to assess differentiation and cell death, and also assess new cell survival using the thymidine analogue EdU. They conclude that diminished swimming behavior reduces neural proliferation but increases over differentiation, and that this effect is dependent upon normal activity in the DRG.

The authors have conducted very thorough and detailed revisions and bring in a considerable amount of new information to confirm the solidity of their methodologies or interpretations. This is a highly interesting and convincing piece of work.

We are very grateful to the Reviewers for their positive feedback regarding the study as a whole.

Please consider the following 3 points:

1) Given the results from (PMID 26001123), which show very clear effects of AG1479 on proliferation in the developing zebrafish brain, the authors should compare their results to this paper in the Discussion section.

We completely agree that this point should be incorporated into the Discussion section of the manuscript. In retrospect, we should have done this in the previous version of the manuscript, as opposed to only limiting our comments to the response letter.

As we discussed in our response to the previous review, the methodology of PMID 26001123 and the work we performed here have significant differences, including a near 8-fold reduction in drug concentration that we used and the focus on different regions of the brain. We believe that we clearly addressed this point in our previous revised manuscript using experiments and new analyses of pre-existing data to demonstrate that early, transient AG1478 treatment had no effect on pallial neurogenesis at 3 dpf or forebrain size by 6 dpf. In the present revised manuscript, we incorporate this comparison with PMID 26001123 in our Discussion section. As before, we believe these experiments, in conjunction with our experiments that do not include AG1478 treatments, collectively suggest that movement-dependent pallial neurogenesis is mediated in part via peripheral neural feedback, likely dorsal root ganglia.

2) Along the same lines, the title may too strongly state that the phenomenon is mediated via the DRG. Given the caveats of AG1479, some revision of the title might be warranted, and so a more "neutral" title, simply mentioning the link between movement and neurogenesis at larval stages, might be appropriate.

Although we designed experiments and have provided additional analyses that suggest AG1478 had no lasting impact on pallial neurogenesis on development prior to movement or Optovin manipulations studied here, we understand that there are some reservations regarding our title. However, we believe simplifying our study title to only mention “movement and neurogenesis in larval zebrafish” ignores the latter half of our experiments, aimed at identifying the modality of sensory feedback mediating this relationship and the isolation of a potential role for DRGs. A simplified title in this fashion would neglect the novelty of our work. As a compromise, we have opted to remove the mention of DRG specifically from the title, instead renaming it: “Movement maintains forebrain neurogenesis via peripheral neural feedback in larval zebrafish”. As our original findings regarding an Optovin + light-induced upregulation of pallial neurogenesis did not involve AG1478 manipulations, our experiments as a whole implicate a role for peripheral neural feedback during movement in forebrain neurogenesis.

I hope you will accept this new title as a reasonable compromise.

3) the manuscript would benefit by providing access to videos of the restricted swimming behavior paradigm, as it would help readers to conceptualize the impact of the mesh barrier on the swimming behavior.

We agree that sample videos of larval movement in our control and restraint paradigms could be useful to readers. We now provide these videos as a supplementary file called Video 1. We reference this video in subsection “Movement restraint reduces swimming episodes without impairing swimming ability” and provide a caption.
